# Supplementary material for: Opportunities and barriers in paediatric pulse oximetry for pneumonia in low-resource clinical settings: a qualitative evaluation from Malawi and Bangladesh
Source: BMJ Open. 2018 Jan 30;8(1):e019177. doi: 10.1136/bmjopen-2017-019177 (PMC5829842; doi:10.1136/bmjopen-2017-019177)
Supplement: Supplementary data [file bmjopen-2017-019177supp001.pdf]

Web Appendix 1 – Topic Guide

- What is your experience of using pulse oximeters in children?
- What have been the main issues you've encountered when using pulse oximeters?
- What have been the things you like most about using the pulse oximeters?
- What type of probes have you used? Have any been better than others? Why?
- Thinking about the probes, we would like to hear your feedback about some aspects of using them: ease of putting and keeping the probe on the child, durability, ease of taking a reading, ease of keeping it clean and storage
- Thinking about the oximeter, we would like to hear your feedback about some aspects of using them: ease of reading the display, durability, battery life and charging, time taken to get a reading
- What things would make the probe and pulse oximeter easier to use?
- What things would make the probe and pulse oximeter harder to use?
