# Supplementary material for: Opportunities and barriers in paediatric pulse oximetry for pneumonia in low-resource clinical settings: a qualitative evaluation from Malawi and Bangladesh
Source: BMJ Open. 2018 Jan 30;8(1):e019177. doi: 10.1136/bmjopen-2017-019177 (PMC5829842; doi:10.1136/bmjopen-2017-019177)
Supplement: Supplementary data [file bmjopen-2017-019177supp002.pdf]

Appendix 2: Summary of pulse oximeter probes presented during focus group discussions with healthcare providers

Probe type	Figure	Product code
Neonatal wrap	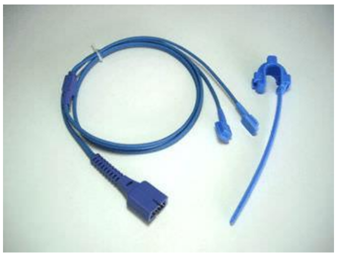	Acare ASYNR-D1
Adult clip	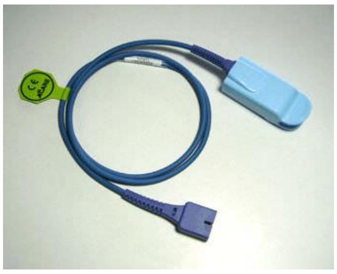	Acare ASANR-D1
Paediatric clip	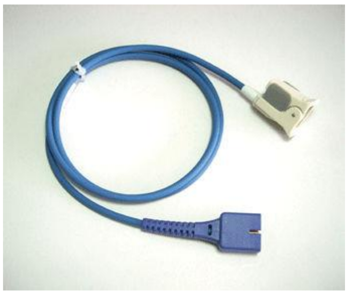	Acare ASPNR-D1
Ear clip	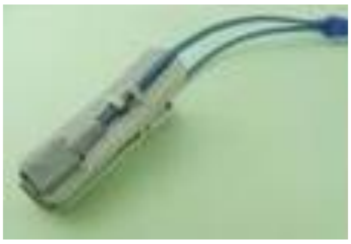	Nellcor U401-2HL
Adult boot	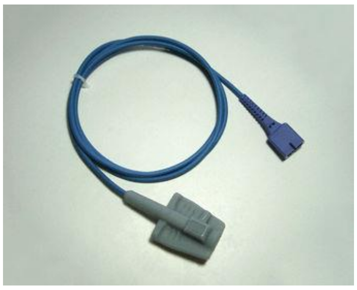	Acare ASSNR-D1
Paediatric boot	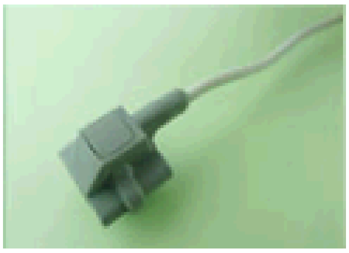	Nellcor U401-2EL
