# Supplementary material for: Opportunities and barriers in paediatric pulse oximetry for pneumonia in low-resource clinical settings: a qualitative evaluation from Malawi and Bangladesh
Source: BMJ Open. 2018 Jan 30;8(1):e019177. doi: 10.1136/bmjopen-2017-019177 (PMC5829842; doi:10.1136/bmjopen-2017-019177)
Supplement: Author's manuscript [file bmjopen-2017-019177.draft_revisions.pdf]

BMJ Open

BMJ Open is committed to open peer review. As part of this commitment we make the peer review history of every article we publish publicly available.

When an article is published we post the peer reviewers' comments and the authors' responses online. We also post the versions of the paper that were used during peer review. These are the versions that the peer review comments apply to.

The versions of the paper that follow are the versions that were submitted during the peer review process. They are not the versions of record or the final published versions. They should not be cited or distributed as the published version of this manuscript.

BMJ Open is an open access journal and the full, final, typeset and author-corrected version of record of the manuscript is available on our site with no access controls, subscription charges or pay-per-view fees (http://bmjopen.bmj.com).

If you have any questions on BMJ Open's open peer review process please email info.bmjopen@bmj.com

Opportunities and barriers in paediatric pulse oximetry for pneumonia in low-resource clinical settings: a qualitative evaluation from Malawi and Bangladesh

Journal:	*BMJ Open*
Manuscript ID	bmjopen-2017-019177
Article Type:	Research
Date Submitted by the Author:	15-Aug-2017

Complete List of Authors:	King, C.; University College London Institute for Global Health,<br>Boyd, Nicholas; Great Ormond Street Hospital For Children NHS Trust, UCL Institute of Child Health<br>Walker, Isabeau; Great Ormond Street Hospital For Children NHS Trust, UCL Institute of Child Health<br>Zadutsa, Beatiwel; Parent and Child Health Initiative<br>Baqui, Abdullah; Johns Hopkins University Bloomberg School of Public Health, International Center for Maternal and Newborn Health<br>Ahmed, Salahuddin; Johns Hopkins University Bloomberg School of Public Health, International Center for Maternal and Newborn Health<br>Islam, Mazharul; Shahjalal University of Science and Technology, Department of Anthropology<br>Kainja, Esther; Parent and Child Health Initiative<br>Nambiar, Bejoy; Institute for Global Health<br>Wilson, Iain; Lifebox Foundation<br>McCollum, Eric ; Johns Hopkins School of Medicine, Eudowood Division of Paediatric Respiratory Diseases; Johns Hopkins University Bloomberg School of Public Health, Department of International Health

**Primary Subject Heading**:	Global health
Secondary Subject Heading:	Paediatrics, Respiratory medicine, Qualitative research, Diagnostics
Keywords:	PAEDIATRICS, QUALITATIVE RESEARCH, Pneumonia, Pulse Oximetry

SCHOLARONE™
Manuscripts

Title: Opportunities and barriers in paediatric pulse oximetry for pneumonia in low-resource clinical settings: a qualitative evaluation from Malawi and Bangladesh

Authors: Carina King[1*], Nicholas Boyd[2], Isabeau Walker[2], Beatiwel Zadutsa[3], Abdullah H. Baqui[4], Salahuddin Ahmed[4], Mazharul Islam[5], Esther Kainja[3], Bejoy Nambiar[1], Iain Wilson[6], Eric D. McCollum[7,8]

Affiliations:

1. Institute for Global Health, University College London, London, UK
2. Great Ormond Street Hospital NHS Foundation Trust, UCL Institute of Child Health, London, UK
3. Parent and Child Health Initiative, Lilongwe, Malawi
4. International Center for Maternal and Newborn Health, Johns Hopkins Bloomberg School of Public Health, Baltimore, USA
5. Department of Anthropology, Shahjalal University of Science and Technology, Sylhet, Bangladesh
6. Lifebox Foundation, London, UK
7. Eudowood Division of Pediatric Respiratory Diseases, Johns Hopkins School of Medicine, Baltimore, USA
8. Department of International Health, Johns Hopkins Bloomberg School of Public Health, Baltimore, USA

***Corresponding author:**

Address: Institute for Global Health, 30 Guilford Street, London, WC1N 1EH; Email: c.king@ucl.ac.uk; telephone: +44 (0) 2076797619

Keywords: Pulse oximeter, oxygen saturation, pneumonia, sub-Saharan Africa, South Asia, children

Word Count: 3,908

For peer review only

Abstract

Objective: To gain an understanding of what challenges pulse oximetry for paediatric pneumonia management poses, how it has changed service provision and what would improve this device for use across paediatric clinical settings in low-income countries.

Design: Focus group discussions (FGDs), with purposive sampling and thematic analysis using a framework approach.

Setting: Community, front line outpatient and hospital outpatient and inpatient settings in Malawi and Bangladesh, which provide paediatric pneumonia care.

Participants: Healthcare providers (HCPs) from Malawi and Bangladesh who had received training in pulse oximetry and had been using oximeters in routine paediatric care, including community healthcare workers, non-physician clinicians or medical assistants, and hospital-based nurses and doctors.

Results: We conducted six FGDs, with 23 participants from Bangladesh and 26 from Malawi. We identified five emergent themes: trust; value; user-related experience; sustainability and design. HCPs discussed the confidence gained through using oximeters, resulting in improved trust from caregivers and valuing the device; although there were conflicts between the weight given to clinical judgement versus oximeter results. HCPs reported the ease of using oximeters, but identified movement and physically smaller children as measurement challenges. Challenges in sustainability related to battery durability and replacement parts, however many HCPs had used the same device longer than four years demonstrating robustness within these settings. Desirable features included back-up power banks and integrated respiratory rate and thermometer capability.

Conclusions: Pulse oximetry was generally deemed valuable by HCPs for use as a spot-check device in a range of paediatric low-income clinical settings. Areas highlighted as challenges by HCPs, and therefore opportunities for re-design, included battery charging and durability, probe fit and sensitivity in paediatric populations.

For peer review only - http://bmjopen.bmj.com/site/about/guidelines.xhtml

Strengths and Limitations

- This is the first study to report on end-user perceptions of opportunities, challenges and desirable design features of pulse oximeters used for paediatric pneumonia management in low-resource settings, including community and outpatient providers.
- Pulse oximeters were valued by healthcare providers, but challenges were highlighted with use in smaller and moving children. Desirable features to improve pulse oximeters for low-resource paediatric settings included improved battery life, integrated respiratory rate and temperature, and quicker results.
- A key strength was the wide range of healthcare provider perspectives included, from community to referral hospital settings in South Asia and sub-Saharan Africa.
- The study was limited to participant's experience of using specific pulse oximeters and therefore may lack generalizability to other paediatric pulse oximeters not used in these settings.

For peer review only

For peer review only - http://bmjopen.bmj.com/site/about/guidelines.xhtml

Introduction

Several interventions, such as Pneumococcal conjugate vaccine (PCV) and standardised guidelines for diagnosis and treatment, have led to reductions in pneumonia morbidity and mortality over the last twenty years [1 2]. However, in spite of these gains, pneumonia remains the leading cause of infectious mortality amongst children globally, with the vast majority of the burden falling in sub-Saharan Africa and south Asia [3]. To accelerate reductions in pneumonia mortality, further refinement of diagnosis and treatment pathways are needed, including correct referral and access to oxygen treatment [4].

Pulse oximetry non-invasively measures peripheral arterial oxyhemoglobin saturation (SpO_2). Hypoxemia (defined as an SpO_2 <90%) is included in the World Health Organization (WHO) guidelines as a pneumonia danger sign [5], and is associated with increased mortality from pneumonia, as well as other illnesses like malaria [6-8]. Recent evidence from Malawi has also indicated that a SpO_2 90-92% is predictive of mortality amongst children hospitalized with pneumonia [8].

While some studies have attempted to predict hypoxemia in children with pneumonia using a combination of clinical signs, there has been mixed success [9-11]. Clinical signs alone fail to identify a proportion of hypoxemic children based on the current WHO guidelines, which results in a missed opportunity for referral and appropriate treatment [12 13]. In addition, the subjectivity of clinical signs can lead to variation in care – especially among community healthcare workers (CHWs), who often lack ongoing supervision.

Pulse oximeters have been successfully used in low-resource paediatric settings, but are yet to be widely adopted, particularly during outpatient care [14 15]. The Ethiopian Ministry of Health has demonstrated leadership in this area, setting up an initiative in 2016 to ensure oximetry and oxygen therapy are available nationally across the healthcare system [16]. However, Ethiopia is an exception, with implementation of oximetry in other developing countries continuing to be slow. Implementation barriers cited include cost, lack of appropriately designed, robust oximeters and universal paediatric probes and issues with training and supervision [17].

In order to better understand current barriers to use of pulse oximetry by healthcare providers (HCPs) in a range of healthcare settings, and explore opportunities that this technology provides, input from end-users is needed [18]. With the ultimate goal of designing a universal paediatric probe for all levels of healthcare services in resource-poor settings, we aimed to gain an understanding of the challenges of pulse oximetry, how its use has changed service provision and how current devices

For peer review only - http://bmjopen.bmj.com/site/about/guidelines.xhtml

could be improved for these settings. This end-user perspective is currently limited in the literature and is essential to ensure investment in pulse oximetry is sustainable and effective.

Methods

We conducted a qualitative study with HCPs from different levels of the healthcare system in Malawi (Mchinji district, central region) and Bangladesh (Sylhet district, northeast region) from May – July 2016, as part of a wider programme of work aiming to design a universal paediatric oximeter probe.

Setting:

In Malawi there are three levels of government provided healthcare: CHWs (locally known as Health Surveillance Assistants), health centres and district hospitals. CHWs conduct weekly or bi-weekly village clinics and home visits providing basic integrated community case management (iCCM) for paediatric infections [19 20]. Health centres are outpatient facilities run by nurses, clinical officers or medical assistants, and District Hospitals have inpatient facilities with capacity for oxygen treatment. In Bangladesh, the study was conducted at Projahnmo, a research consortium comprised of Johns Hopkins University and several local non-governmental organizations in partnership with the Bangladesh Ministry of Family Health and Welfare. Current Projahnmo activities are integrated within three government-operated sub-district hospitals, called Upazila Health Complexes (UHCs), and the referral government hospital in Sylhet city (Osmani Medical College), all of which are staffed by physicians and nurses. The UHCs operate outpatient clinics for children under five and provide basic inpatient paediatric care, including oxygen. The majority of government provided inpatient care is provided at Osmani Medical College. Female CHWs employed by Projahnmo conduct bimonthly household surveillance, with a subset of CHWs providing weekly surveillance as a part of a PCV effectiveness study. Projahnmo CHWs conduct basic clinical assessments and refer ill children for care at the UHCs; they do not administer medicines themselves.

Currently pulse oximetry is not part of standard care in the community or health centre setting in either Malawi or Bangladesh. In Mchinji, pulse oximetry was successfully introduced into all three healthcare settings in 2012 as part of a PCV research project, using the Acare Technology AH-MX manufactured Lifebox® oximeter and universal adult clip probe[12]. Since 2015, a National Institutes of Health-funded study (K01TW009988) trained and supplied all Projahnmo clinical staff in Bangladesh, including CHWs, with pulse oximeters to screen children for hypoxemia, using the Masimo Rad5® oximeter and the LNCS® Y-I Multisite wrap probe (Figure 1).

For peer review only - http://bmjopen.bmj.com/site/about/guidelines.xhtml

Design:

We conducted focus group discussions (FGDs). We planned three FGDs in each country, aiming to recruit between 6 and 10 people for each FGD (up to 60 participants in total). The groups were planned to be CHWs, health centre or UHC staff, and referral hospital staff separately. Conducting separate FGDs for the different types of healthcare workers was to allow context-specific discussions and encourage participants with varying training backgrounds to feel confident about raising challenges relevant to their specific setting.

Sampling:

HCPs were purposefully sampled from sites where pulse oximetry had been introduced, and the participants had received some form of training or mentorship in oximetry. Participants were identified by local researchers (BZ in Malawi and SA in Bangladesh) to be a representative sample of HCPs from their setting (e.g. small and large health centres, inpatient wards and outpatient departments in the hospital), and contacted directly by phone. All HCPs contacted participated. Participants were reimbursed for their travel costs and provided with refreshments.

Procedure:

FGDs were led by local researchers with experience in conducting qualitative research, with support from a facilitator with knowledge of pulse oximeters. The FGDs were divided into two sections, the first addressing the participants' personal experience with using pulse oximeters. The topic guide included: positive and negative experiences, and possible improvements and challenges (Web appendix 1). During the second part of the discussion, the participants were presented with different probe designs and given an opportunity to use them for an hour. Following this, the discussion addressed positive and negative aspects of the different designs to encourage critical thinking of possible design solutions to the current limitations of a universal paediatric probe.

The FGDs were audio recorded and then transcribed. The participants were told to answer in English or their native tongue (Chichewa, Bangla or Sylheti), depending on their preference and ease of expressing concepts. Recordings were transcribed and translated where necessary. Translations for Malawi were done by BZ and EK together until final transcripts were agreed, and by an independent professional service for Bangladesh.

Analysis:

We analysed the data thematically using a framework approach, as an appropriate method for a multi-disciplinary team conducting health research [21]. This process involves five steps:

For peer review only - http://bmjopen.bmj.com/site/about/guidelines.xhtml

familiarisation, identifying a thematic framework, indexing, mapping and interpretation [22]. The transcripts and notes from the FGDs were printed and coded on paper, with the coding matrix created in Microsoft Excel. CK and KF independently familiarised themselves and indexed the data, and the emergent themes were discussed until a consensus was reached on the mapping and interpretation of the data. This interpretation was shared with the local researchers (BZ and EK in Malawi; EDM and MI in Bangladesh) for further discussion until all were in agreement.

Ethics:

Written informed consent was obtained from all FGD participants. This study was reviewed and approved by the University College London research ethics committee (8075/003), Johns Hopkins Medicine Institutional Review Board (IRB00047406), the Malawi National Health Sciences Research Committee (16/4/1570) and Bangladesh Medical Research Council (BMRC/NREC/2013-2016/1272).

Results

We conducted six FGDs, with 23 participants from Bangladesh and 26 from Malawi (Table 1). We identified five emergent themes: trust; value; user-related experience; sustainability and design.

Trust

Trust emerged as a theme both in terms of how the HCPs interpret the oximetry results, and how caregivers interact with HCPs and the pulse oximeter. We found that all cadres of HCPs in both sites had an awareness of the fallibility of the oximetry readings, specifically relating to lower SpO_2 values. For SpO_2 levels which were deemed abnormal, <90% up to <95% according to different participants, HCPs stated that they would often re-check the result before making a referral or treatment decision:

"if we see it is 89% we change the probe or change the finger" (Hospital, Bangladesh)

However, questioning the validity of these lower SpO_2 results in the context of a child's clinical condition was only discussed by the HCPs who worked in the hospital setting. This difference in the trust placed in the SpO_2 results by different types of HCPs suggests that more in-depth clinical training and understanding of the technology may result in different clinical applications:

"sometimes the pulse oximeter can give readings which you are not comfortable with according to the presentation of the child […] most of the time when it happens like that, we just use our judgement" (Hospital, Malawi)

For peer review only - http://bmjopen.bmj.com/site/about/guidelines.xhtml

An outcome of using pulse oximeters for pneumonia diagnosis was a change in parental and community understanding and perceptions of care, with HCPs discussing increased trust in their referral and treatment decisions. This worked in two ways, firstly with the oximeter acting as a direct feedback and education tool:

"if the mother is able to read you can show the exact figure and she will accept the treatment of oxygen, [before] it was very difficult to explain the role or the importance of the oxygen machine and some mothers refused" (Hospital, Malawi)

Secondly, in Malawi HCPs projected that the oximeters had improved clinical care, and therefore outcomes, which led caregivers to be more inclined to accept the referral or treatment being recommended, especially in the case of oxygen:

"[previously] in the village they were saying that when a child is put on the oxygen machine it facilitates death, therefore it was making problems, but this time because children are put on oxygen earlier they survive, it's because we knew the saturation" (Health centre, Malawi)

Value

The theme of value relates to the inherent value of improved decision making, HCPs perceived self-value (i.e. confidence) in their clinical work, and the physical value placed on maintaining a working pulse oximeter. As pneumonia is classified using a range of non-specific and often subjective clinical signs, HCPs discussed the positive addition of this more objective measure:

"…by looking at this we can understand how much respiratory distress is in there. Of course this helps us a lot." (Health Centre, Bangladesh)

In both sites HCPs from frontline settings (CHWs, health centres and UHCs) stated that the pulse oximeters had changed the way they work and given them confidence in making referral decisions. Interestingly however, in the referral hospital setting in Bangladesh where staff training is higher, very little value was placed on the pulse oximeter for improving their clinical performance, with the ability to conduct chest x-rays, lung ultra-sound and their clinical judgement valued more highly:

"…its sensitivity and specificity is very negligible to be taken as a diagnostic tool." (Hospital, Bangladesh)

In Bangladesh the CHWs reported pride in using the oximeters. In Malawi, the CHWs (who individually own the oximeters) placed a physical value on the oximeters and discussed the personal effort, such as paying out of pocket for charging, they put in to maintaining a working device:

For peer review only - http://bmjopen.bmj.com/site/about/guidelines.xhtml

"…we have been trying all that is humanly possible to take care of these things, but it only becomes a problem when it comes to the issue of charging." (CHW, Malawi)

This was also reflected at the health centre, where not all facilities have electricity and one or two staff are responsible for assessing children; at the referral hospital however this was not discussed, with oximeters belonging to the ward, which has a more consistent power supply. Ward-based ownership was discussed as a challenge by hospital staff, suggesting individual ownership could result in improved care and maintenance.

"…some of the clinicians do not take care of them, so when the machine is not working it means the whole department is affected" (Hospital, Malawi)

User-related experience

HCPs at all levels discussed their experiences of using pulse oximeters in children under 5-years, presenting challenges, their solutions and perceptions of usability. The time taken to get a measurement ranged widely, with CHWs in Bangladesh agreeing measurements took less than 1 minute but in Malawi that it could take up to 20 minutes. The factors that increased the time taken to get a measurement were consistently cited as movement and physically smaller children, and in Malawi dirty toes making measurements difficult:

"Getting readings from irritable babies is a bit tough and it takes time." (Health centre, Bangladesh)

"…using it on a child up to six months of age, sometimes it has been a problem because these children have got small fingers, so although we use toes sometimes they are also small and the child is afraid so they start crying. So we have got other things we can give a child to play with but it is a little bit of a problem, but at the end we get the results." (CHW, Malawi)

Solutions to these challenging children included asking caregivers to breastfeed, giving them a toy to distract them, and simply waiting. The term used frequently to describe challenging children was 'fear', with the HCPs stating that children are afraid of having the measurements taken and even that the sensors' red light caused this fear. Despite these issues in small and agitated infants, the oximeters were considered easy to use:

"…it's not complicated, it doesn't need complicated education for a healthcare worker to use, with a good explanation from a colleague or friend you are able to use it." (Hospital, Malawi)

For peer review only - http://bmjopen.bmj.com/site/about/guidelines.xhtml

For peer review only

There was also the acknowledgement that time to reading was not as important as getting the correct measurement; for some respondents, the reason some measurements take longer is the desire to get a reliable reading. This included cleaning the child's digits or repositioning the probe:

"…taking longer does not mean that one doesn't know the procedure, but sometime it's because you want to give the correct reading." (CHW, Malawi)

A key challenge reported by frontline HCPs in Malawi was around keeping the oximeter charged; this was not considered a significant challenge in Bangladesh. However, here they had issues with ensuring the oximeter remained dry and protected during rains and being fully waterproof was desirable. Depending on usage, battery life was reported as 1 week – 2 months. In Malawi, none of the CHWs and only some of the health centres have access to electricity for charging the re-usable batteries, and therefore they reported travelling to use commercial charging services:

"Most of the times we take the pulse oximeter to the trading centre and charge it, then we pay for that only to make sure that we are working, but sometimes you also feel you become bankrupt." (Health centre, Malawi)

<u>Sustainability</u>

Sustainability was discussed in terms of the device's durability, and the need for continued professional development. Generally the pulse oximeters were thought of as robust and durable, with some of the HCPs having used their device for over four years without replacements. However, the battery was highlighted as the least durable part of the device, and there was a perception that when the battery was worn down the readings became less reliable.

"There is a matter with the battery too, if the battery is not enough the reading takes a long time to appear. It sometimes gives false negative readings." (Hospital, Bangladesh)

This related to the HCPs suggestion of having on-going maintenance support rather than wanting replacement devices. HCPs described the need for on-going training and support, but also expressed a desire for more in-depth education on how oximetry works which goes beyond the basic training to take a reliable measurement:

"A person gets used to what they are doing once they have been oriented. I think sometimes it's also good for you and your team to orient us on how this thing works [...] the way this thing works, we don't know" (Health centre, Malawi)

For peer review only - http://bmjopen.bmj.com/site/about/guidelines.xhtml

In terms of keeping the devices clean, we found conflicting opinions between Malawi and Bangladesh. Malawi deemed the probes easy to clean and store securely, although the light colour and materials of the device was thought to show dirt easily. However, in Bangladesh cleaning was described as burdensome; this likely reflects the different devices and therefore methods needed for cleaning, or different perceptions of the importance and frequency of cleaning.

"It is hard work to clean it with hexsol and cotton after coming back from the field every day. If we could get something else to clean it with so that we can clean once a week, I don't like cleaning it every day." (CHW, Bangladesh)

<u>Design</u>

The key challenges mentioned repeatedly across sites and HCP cadres were the battery, sensitivity of the probe in relation to movement or low perfusion, and the probe fit in younger children. Box 1 summarises the design features requested or suggested to improve the pulse oximeters for use in these low-resource settings.

The oximeters which HCPs used were designed for continuous monitoring; therefore oxygen saturation is not a single static result. This was seen as a negative, with HCPs in both sites wanting the ability to stop at a result and even store measurements (e.g. a blood glucose monitor):

"Readings would fluctuate if the baby moves. We don't want that. After getting the actual reading it should stay fixed." (CHW, Bangladesh)

In Bangladesh specifically, the CHWs stated a preference for numbers or bars to indicate measurement quality, rather than a dynamic waveform display. This opinion was not reflected in Malawi, which could be a result of using different devices or different training. A specific challenge presented by CHWs in Malawi was the use of the oximeter in direct sunlight; CHWs often hold clinics outside and they faced the combined challenges of bright sunlight and dust, both of which they reported as challenges in taking measurements.

"…it returns the correct results when you are in the shade, but while you are in sunlight it fails to determine good results." (CHW, Malawi)

Positive design features included the portability of devices, the ease of using them and perceived durability, with little direct criticism of the oximeters that the HCPs had been using:

"…of the things I like most about using the pulse oximeter, the first one is the portability, because I can use it anywhere." (Hospital, Malawi)

For peer review only - http://bmjopen.bmj.com/site/about/guidelines.xhtml

Discussion

We investigated end-user experiences of using pulse oximeters by a range of different HCPs across clinical settings in Malawi and Bangladesh. The FGDs highlighted similarities in experience, such as challenges in battery durability, the difficulty of small and agitated children and the positive impact of oximeters on clinical practice. However, there were key differences between Malawi and Bangladesh and between HCP cadres.

Of note was the difference in perceived ease of cleaning, which was seen as more burdensome in Bangladesh. This is likely associated with the Y-shaped wrap probe design, compared to the more easily cleaned clip design used in Malawi (Figure 1). Interestingly though, most critiques were similar between sites, highlighting some of the major challenges of using pulse oximetry in children – namely movement, low perfusion and small digits. This consistency between sites suggests these challenges are not device dependent and therefore a specifically designed re-usable device for universal paediatric use in low-resource settings is needed.

We identified differences in the sense of value placed on the oximeters by HCPs, with the higher trained HCPs attributing less value to the results than the HCPs with more limited training. Those with more training valued their clinical judgement more and were more willing to question the accuracy of SpO_2 results. This poses interesting lessons for scaling-up implementation and training, as despite perceptions that obtaining a SpO_2 measurement is generally easy, the interpretation of the result is more nuanced. Sustained mentorship and more in-depth training were desired by the HCPs, and this needs to be considered as part of any implementation programme.

As the oximeters were used as spot-check devices rather than continuous monitors, as would generally be found in operating theatres or high-dependency care in high-income settings, many of the suggested design changes related to improving the devices for this process. One example of this was the need for improved battery-life and charging, with HCPs highlighting their limited ability to easily access charging points, unlike high-income inpatient settings. Consistently, the desire for quicker, static results and a movement tolerant probe with improved fit on younger infants was important. Unexpected issues, such as usability in direct sunlight, emphasize the importance of end-user engagement in product development as clinical devices designed for high-income settings would not need to be robust to outdoor use.

The idea of a pulse oximeter being able to improve trust between a caregiver and healthcare provider poses potentially exciting opportunities for improving referral and treatment for paediatric

For peer review only - http://bmjopen.bmj.com/site/about/guidelines.xhtml

pneumonia. Early diagnosis and treatment as downstream in the health system as possible, ideally to the level of CHWs, are key strategies for improving pneumonia outcomes and therefore reducing morbidity and mortality burden [23]. Therefore, an objective and simple clinical tool with in-built decision support, e.g. auditory or visual alarms when the SpO_2 is outside of normal range, presents an opportunity for caregiver education and empowerment in the referral decision-making process. Recent data from Malawi supports this notion. Frontline Malawian HCPs using pulse oximeters during routine outpatient care demonstrated that among children with pneumonia who were clinically eligible for referral, children with a SpO_2<90% were more than twice as likely to have been correctly referred compared to those with a $SpO_2 \geq 90\%$ [12]. Interestingly, this has not necessarily been the case with other more objective diagnostic tools, with examples of rapid diagnostic malaria tests leading to provider-caregiver tensions around treatments [24 25].

This study was potentially subject to social-desirability bias, with healthcare workers expressing opinions which they thought the facilitators wanted to hear. The purpose of the study was explained to the participants during the consent process, and was highlighted as an opportunity for them to contribute to the design of a revised paediatric oximeter and probe. To mitigate this potential bias, the facilitators encouraged the participants to be critical throughout, and the FGDs were conducted amongst peers, rather than between different HCP cadres with different educational backgrounds and social dynamics. Both positive and negative views were given in both Malawi and Bangladesh, and by different types of HCPs, therefore we do not feel this bias is likely to have impacted our findings.

Overall pulse oximeters were valued by HCPs, despite challenges with charging, maintenance and speed of achieving accurate readings in moving or smaller children. This implies that making improvements to currently available oximeters and probes could further facilitate successful implementation of this technology at the community through to the hospital level for routine paediatric care. Based on these data, we recommend that efforts to re-design a pulse oximeter for paediatric spot-checks focus on improvements to battery durability, better fit for smaller digits and the speed at which readings are obtained; these were all important challenges which did not necessarily have local solutions presented. More substantive design changes could focus on alternative power and charging systems (e.g. solar charging) and '3-in-1' devices which include respiratory rate and temperature measurements.

For peer review only - http://bmjopen.bmj.com/site/about/guidelines.xhtml

Acknowledgments

We would like to thank all those healthcare providers who took part in the focus group discussions for their time. We would like to acknowledge other members of the study team: Katie Fernandez (Lifebox); Charles Makwenda and Tambosi Phiri (Parent and Child Health Initiative); Tim Colbourn (University College London); Mike Bernstein (Physio Monitor); and Nazma Begum, Arun Dutta Roy and Abu Abdullah Mohammad Hanif (Johns Hopkins University-Bangladesh).

Funding

This study was funded by the Bill & Melinda Gates Foundation (grant number: OPP1133291).

Conflict of Interest

Authors declare no conflicts of interest.

Author contributions

The qualitative study was designed and topic guides developed by IWi, IWa, CK and EDM, and the field manual written by CK. In Malawi, BZ and EK arranged, conducted, transcribed and translated the FGDs. In Bangladesh SA and MI arranged and conducted the FGDs. The data was coded and analysed by CK and KF. The manuscript was written by CK, with considerable input from KF and EDM. All authors read, commented and approved the manuscript.

Data Sharing Statement

Anonymised transcripts can be shared, following the signing of a data sharing agreement, subject to approval from the relevant National ethics committees. For further information please contact Dr. Carina King: c.king@ucl.ac.uk.

For peer review only

References

1. Sazawal S, Black RE, Pneumonia Case Management Trials G. Effect of pneumonia case management on mortality in neonates, infants, and preschool children: a meta-analysis of community-based trials. Lancet Infect Dis 2003;**3**(9):547-56.
2. Niessen LW, ten Hove A, Hilderink H, et al. Comparative impact assessment of child pneumonia interventions. Bull World Health Organ 2009;**87**(6):472-80.
3. Walker CL, Rudan I, Liu L, et al. Global burden of childhood pneumonia and diarrhoea. Lancet 2013;**381**(9875):1405-16.
4. Organisation WH. *Oxygen therapy for children: a manual for health workers*. Geneva: WHO, 2016.
5. . WHO Recommendations on the Management of Diarrhoea and Pneumonia in HIV-Infected Infants and Children: Integrated Management of Childhood Illness (IMCI). Geneva, 2010.
6. Orimadegun A, Ogunbosi B, Orimadegun B. Hypoxemia predicts death from severe falciparum malaria among children under 5 years of age in Nigeria: the need for pulse oximetry in case management. Afr Health Sci 2014;**14**(2):397-407.
7. Chhibber AV, Hill PC, Jafali J, et al. Child Mortality after Discharge from a Health Facility following Suspected Pneumonia, Meningitis or Septicaemia in Rural Gambia: A Cohort Study. Plos One 2015;**10**(9):e0137095.
8. Hooli S, Colbourn T, Lufesi N, et al. Predicting Hospitalised Paediatric Pneumonia Mortality Risk: An External Validation of RISC and mRISC, and Local Tool Development (RISC-Malawi) from Malawi. Plos One 2016;**11**(12):e0168126.
9. Wandeler G, Pauchard JY, Zangger E, et al. Which clinical signs predict hypoxaemia in young Senegalese children with acute lower respiratory tract disease? Paediatrics and International Child Health 2015;**35**(1):65-68.
10. Basnet S, Adhikari RK, Gurung CK. Hypoxemia in children with pneumonia and its clinical predictors. The Indian Journal of Pediatrics 2006;**73**(9):777-81.
11. Lodha R, Bhadauria PS, Kuttikat AV, et al. Can Clinical Symptoms or Signs Accurately Predict Hypoxemia in Children with Acute Lower Respiratory Tract Infections? Indian Pediatr 2003;**41**:129-35.
12. McCollum ED, King C, Deula R, et al. Pulse oximetry for children with pneumonia treated as outpatients in rural Malawi. Bull World Health Organ 2016;**94**:893-902.
13. Duke T, Subhi R, Peel D, et al. Pulse oximetry: technology to reduce child mortality in developing countries. Ann Trop Paediatr 2009;**29**(3):165-75.

For peer review only

For peer review only - http://bmjopen.bmj.com/site/about/guidelines.xhtml

14. Emdin CA, Mir F, Sultana S, et al. Utility and feasibility of integrating pulse oximetry into the routine assessment of young infants at primary care clinics in Karachi, Pakistan: a cross-sectional study. BMC Pediatrics 2015;**15**(1):141.

15. King EM, Lieu C, Kasasa A, et al. Pulse Oximetry as a Screening Tool to Detect Hypoxia Associated with Early-onset Sepsis in Asymptomatic Newborns: A Feasibility Study in a Low-income Country. British Journal of Medicine & Medical Research 2014;**4**(5):1115-28.

16. Ginsburg AS, Izadnegahdar R, Klugman KP. World Pneumonia Day 2016: pulse oximetry and oxygen. The Lancet Global Health 2016;**4**(12):e893-e94.

17. Ginsburg AS, Van Cleve WC, Thompson MI, et al. Oxygen and pulse oximetry in childhood pneumonia: a survey of healthcare providers in resource-limited settings. J Trop Pediatr 2012;**58**(5):389-93.

18. Spence H, Baker K, Wharton-Smith A, et al. Childhood pneumonia diagnostics: community health workers' and national stakeholders' differing perspectives of new and existing aids. Glob Health Action 2017;**10**(1):1290340.

19. Nsona H, Mtimuni A, Daelmans B, et al. Scaling up Integrated Community Case Management of Childhood Illness: Update from Malawi. Am J Trop Med Hyg 2012;**87**(Suppl 5):54-60.

20. Malalwi Go. Integrated Management of Childhood Illness - Caring for Newborns and Children in the Community: Manual for Healt hSurveillance Assistants. In: Health Mo, ed. Lilongwe, Malawi, 2008.

21. Gale NK, Heath G, Cameron E, et al. Using the framework method for the analysis of qualitative data in multi-disciplinary health research. BMC Medical Research Methodology 2013;**13**(1):117.

22. Ritchie J, Spencer L. Qualitative data analysis for applied policy research. The qualitative researcher's companion 2002;**573**:305-29.

23. McCollum ED, King C, Hammitt LL, et al. Reduction of childhood pneumonia mortality in the Sustainable Development era. Lancet Respir Med 2016;**4**(12):932-33.

24. Ansah EK, Reynolds J, Akanpigbiam S, et al. "Even if the test result is negative, they should be able to tell us what is wrong with us": a qualitative study of patient expectations of rapid diagnostic tests for malaria. Malaria Journal 2013;**12**(1):258.

25. Hutchinson E, Reyburn H, Hamlyn E, et al. Bringing the state into the clinic? Incorporating the rapid diagnostic test for malaria into routine practice in Tanzanian primary healthcare facilities. Glob Public Health 2015:1-15.

For peer review only

For peer review only - http://bmjopen.bmj.com/site/about/guidelines.xhtml

Table 1: Summary of the FGD participants and their work experience

	Community level	Health centre or Upazila Health Complex	Hospital
**BANGLADESH**			
Total participants	8	7	8
Job titles (number)	Community healthcare worker (8)	Physician (4)<br>Medical officer (3)	Senior staff nurse (1)<br>Associate professor (2)<br>ICU staff (1)<br>Anaesthesiologist (1)<br>Assistant registrar (1)<br>Intern medical officer (1)
Work experience (mean, range)	1.7 years (0.6 – 4)	2.3 years (1 – 6)	14.7 years (0.5 – 32)
**MALAWI**			
Total participants	9	8	9
Job titles	Community healthcare worker (8)<br>Vital signs assistant (1)	Medical assistant (7)<br>Medical technician (1)	Clinical officer (3)<br>Nurse midwife (3)<br>Medical assistant (3)
Years' work experience (mean)	10.6 years (5 – 20)	8.3 years (3 – 23)	8.1 years (4 – 13)

For peer review only

For peer review only - http://bmjopen.bmj.com/site/about/guidelines.xhtml

463 **Panel 1:** Suggestions of desirable features or improvements

**Challenge:**	**Design suggestion:**
Probe fit	Supplied with multiple sizes of probes for different ages<br>A single cable with multiple probes that can be changed (e.g. clipped into the cable)<br>Softer material for a more comfortable fit
Probe placement	Probe made of transparent material so sensor placement on the nail can be seen
Cleaning	Alcohol wipes provided for easier cleaning<br>Different colour probe to make it easier to see the dirt, but does not look dirty quickly
Power	Solar powered charger with rechargeable batteries<br>Back-up power bank<br>Supplied with a spare battery
Agitated children	Toy feature in the device to distract the child<br>Improve the sensitivity of the device to be quicker<br>Improve the sensitivity of the device to tolerate movement
Integrated spot-check device	Store results in a memory that can later be accessed<br>Static oxygen saturation result display<br>'3-in-1' device which includes temperature and respiratory rate measurements as well<br>Shorter cable length for easier portability

464

For peer review only - http://bmjopen.bmj.com/site/about/guidelines.xhtml

Figure 1: Pulse oximeters and probe used by healthcare providers in routine clinical care

a. Lifebox® oximeter and adult universal clip probe used in Malawi (accessed on 1st July 2017 from: www.lifebox.org)

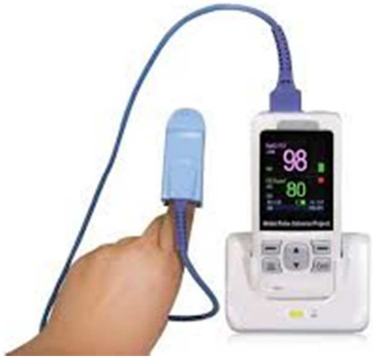

b. Masimo Rad5® oximeter and LNCS® Y-I Multisite wrap probe used in Bangladesh (accessed on 1st July 2017 from: www.pacificmedicalsupply.com)

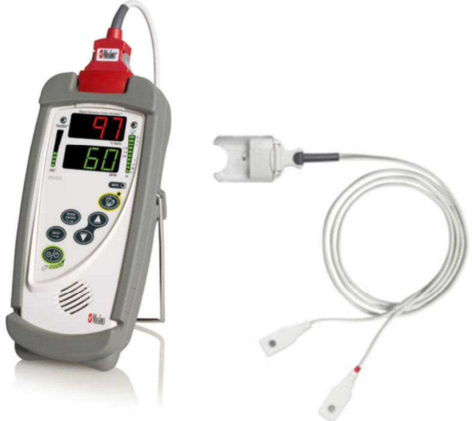

For peer review only

Web Appendix 1 – Topic Guide

- What is your experience of using pulse oximeters in children?
- What have been the main issues you've encountered when using pulse oximeters?
- What have been the things you like most about using the pulse oximeters?
- What type of probes have you used? Have any been better than others? Why?
- Thinking about the probes, we would like to hear your feedback about some aspects of using them: ease of putting and keeping the probe on the child, durability, ease of taking a reading, ease of keeping it clean and storage
- Thinking about the oximeter, we would like to hear your feedback about some aspects of using them: ease of reading the display, durability, battery life and charging, time taken to get a reading
- What things would make the probe and pulse oximeter easier to use?
- What things would make the probe and pulse oximeter harder to use?

For peer review only

BMJ Open

Opportunities and barriers in paediatric pulse oximetry for pneumonia in low-resource clinical settings: a qualitative evaluation from Malawi and Bangladesh

Journal:	*BMJ Open*
Manuscript ID	bmjopen-2017-019177.R1
Article Type:	Research
Date Submitted by the Author:	10-Oct-2017
Complete List of Authors:	King, C.; University College London Institute for Global Health,<br>Boyd, Nicholas; Great Ormond Street Hospital For Children NHS Trust, UCL Institute of Child Health<br>Walker, Isabeau; Great Ormond Street Hospital For Children NHS Trust, UCL Institute of Child Health<br>Zadutsa, Beatiwel; Parent and Child Health Initiative<br>Baqui, Abdullah; Johns Hopkins University Bloomberg School of Public Health, International Center for Maternal and Newborn Health<br>Ahmed, Salahuddin; Johns Hopkins University Bloomberg School of Public Health, International Center for Maternal and Newborn Health<br>Islam, Mazharul; Shahjalal University of Science and Technology, Department of Anthropology<br>Kainja, Esther; Parent and Child Health Initiative<br>Nambiar, Bejoy; Institute for Global Health<br>Wilson, Iain; Lifebox Foundation<br>McCollum, Eric ; Johns Hopkins School of Medicine, Eudowood Division of Paediatric Respiratory Diseases; Johns Hopkins University Bloomberg School of Public Health, Department of International Health
**Primary Subject Heading**:	Global health
Secondary Subject Heading:	Paediatrics, Respiratory medicine, Qualitative research, Diagnostics
Keywords:	Pulse oximeter, Oxygen saturation, Pneumonia, Children, Sub-Saharan Africa, South Asia

SCHOLARONE™
Manuscripts

Title: Opportunities and barriers in paediatric pulse oximetry for pneumonia in low-resource clinical settings: a qualitative evaluation from Malawi and Bangladesh

Authors: Carina King[1*], Nicholas Boyd[2], Isabeau Walker[2], Beatiwel Zadutsa[3], Abdullah H. Baqui[4], Salahuddin Ahmed[4], Mazharul Islam[5], Esther Kainja[3], Bejoy Nambiar[1], Iain Wilson[6], Eric D. McCollum[7,8]

Affiliations:

1. Institute for Global Health, University College London, London, UK
2. Great Ormond Street Hospital NHS Foundation Trust, UCL Institute of Child Health, London, UK
3. Parent and Child Health Initiative, Lilongwe, Malawi
4. International Center for Maternal and Newborn Health, Johns Hopkins Bloomberg School of Public Health, Baltimore, USA
5. Department of Anthropology, Shahjalal University of Science and Technology, Sylhet, Bangladesh
6. Lifebox Foundation, London, UK
7. Eudowood Division of Pediatric Respiratory Diseases, Johns Hopkins School of Medicine, Baltimore, USA
8. Department of International Health, Johns Hopkins Bloomberg School of Public Health, Baltimore, USA

***Corresponding author:**

Address: Institute for Global Health, 30 Guilford Street, London, WC1N 1EH; Email: c.king@ucl.ac.uk; telephone: +44 (0) 2076797619

Keywords: Pulse oximeter, oxygen saturation, pneumonia, sub-Saharan Africa, South Asia, children

Word Count: 4,039

For peer review only

For peer review only - http://bmjopen.bmj.com/site/about/guidelines.xhtml

Abstract

Objective: To gain an understanding of what challenges pulse oximetry for paediatric pneumonia management poses, how it has changed service provision and what would improve this device for use across paediatric clinical settings in low-income countries.

Design: Focus group discussions (FGDs), with purposive sampling and thematic analysis using a framework approach.

Setting: Community, front line outpatient and hospital outpatient and inpatient settings in Malawi and Bangladesh, which provide paediatric pneumonia care.

Participants: Healthcare providers (HCPs) from Malawi and Bangladesh who had received training in pulse oximetry and had been using oximeters in routine paediatric care, including community healthcare workers, non-physician clinicians or medical assistants, and hospital-based nurses and doctors.

Results: We conducted six FGDs, with 23 participants from Bangladesh and 26 from Malawi. We identified five emergent themes: trust; value; user-related experience; sustainability; and design. HCPs discussed the confidence gained through using oximeters, resulting in improved trust from caregivers and valuing the device; although there were conflicts between the weight given to clinical judgement versus oximeter results. HCPs reported the ease of using oximeters, but identified movement and physically smaller children as measurement challenges. Challenges in sustainability related to battery durability and replacement parts were reported, however many HCPs had used the same device longer than four years demonstrating robustness within these settings. Desirable features included back-up power banks and integrated respiratory rate and thermometer capability.

Conclusions: Pulse oximetry was generally deemed valuable by HCPs for use as a spot-check device in a range of paediatric low-income clinical settings. Areas highlighted as challenges by HCPs, and therefore opportunities for re-design, included battery charging and durability, probe fit and sensitivity in paediatric populations.

For peer review only - http://bmjopen.bmj.com/site/about/guidelines.xhtml

Strengths and Limitations

- This is the first study to report on end-user perceptions of opportunities, challenges and desirable design features of pulse oximeters used for paediatric pneumonia management in low-resource settings, including community and outpatient providers.
- A key strength was the wide range of healthcare provider perspectives included, from community to referral hospital settings in South Asia and sub-Saharan Africa.
- The study was limited to participant’s experience of using specific pulse oximeters and therefore may lack generalizability to other paediatric pulse oximeters not used in these settings.

For peer review only

For peer review only - http://bmjopen.bmj.com/site/about/guidelines.xhtml

Introduction

Several interventions, such as Pneumococcal conjugate vaccine (PCV) and standardised guidelines for diagnosis and treatment, have led to reductions in pneumonia morbidity and mortality over the last twenty years [1 2]. However, in spite of these gains, pneumonia remains the leading cause of infectious mortality amongst children globally, with the vast majority of the burden falling in sub-Saharan Africa and south Asia [3]. To accelerate reductions in pneumonia mortality, further refinement of diagnosis and treatment pathways are needed, including correct referral and access to oxygen treatment [4].

Pulse oximetry non-invasively measures peripheral arterial oxyhemoglobin saturation (SpO_2). Hypoxemia (defined as an SpO_2 <90%) is included in the World Health Organization (WHO) guidelines as a pneumonia danger sign [5], and is associated with increased mortality from pneumonia, as well as other illnesses like malaria [6-8]. Recent evidence from Malawi has also indicated that a SpO_2 90-92% is predictive of mortality amongst children hospitalized with pneumonia [8].

While some studies have attempted to predict hypoxemia in children with pneumonia using a combination of clinical signs, there has been mixed success [9-11]. Clinical signs alone fail to identify a proportion of hypoxemic children based on the current WHO guidelines, which results in a missed opportunity for referral and appropriate treatment [12 13]. In addition, the subjectivity of clinical signs can lead to variation in care – especially among community healthcare workers (CHWs), who often lack ongoing supervision.

Pulse oximeters have been successfully used in low-resource paediatric settings, but are yet to be widely adopted, particularly during outpatient care [14 15]. The Ethiopian Ministry of Health has demonstrated leadership in this area, setting up an initiative in 2016 to ensure oximetry and oxygen therapy are available nationally across the healthcare system [16]. However, Ethiopia is an exception, with implementation of oximetry in other developing countries continuing to be slow. Implementation barriers cited include cost, lack of appropriately designed, robust oximeters and universal paediatric probes and issues with training and supervision [17].

In order to better understand current barriers to use of pulse oximetry by healthcare providers (HCPs) in a range of healthcare settings, and explore opportunities that this technology provides, input from end-users is needed [18]. With the ultimate goal of designing a universal paediatric probe for all levels of healthcare services in resource-poor settings, we aimed to gain an understanding of the challenges of pulse oximetry, how its use has changed service provision and how current devices

For peer review only - http://bmjopen.bmj.com/site/about/guidelines.xhtml

could be improved for these settings. This end-user perspective is currently limited in the literature and is essential to ensure investment in pulse oximetry is sustainable and effective.

Methods

We conducted a qualitative study with HCPs from different levels of the healthcare system in from one site in Malawi (Mchinji district, central region) and one in Bangladesh (Sylhet district, northeast region) from May – July 2016, as part of a wider programme of work aiming to design a universal paediatric oximeter probe.

<u>Setting:</u>

In Malawi there are three levels of government provided healthcare: CHWs (locally known as Health Surveillance Assistants), health centres and district hospitals. CHWs conduct weekly or bi-weekly village clinics and home visits providing basic integrated community case management (iCCM) for paediatric infections [19 20]. Health centres are outpatient facilities run by nurses, clinical officers or medical assistants, and District Hospitals have inpatient facilities with capacity for oxygen treatment. In Mchinji, pulse oximetry was successfully introduced into all three healthcare settings in 2012 as part of a PCV research project, using the Acare Technology AH-MX manufactured Lifebox® oximeter and universal adult clip probe (Figure 1a) [12].

In Bangladesh, the study was conducted at Projahnmo, a research consortium comprised of Johns Hopkins University and several local non-governmental organizations in partnership with the Bangladesh Ministry of Family Health and Welfare. Current Projahnmo activities are integrated within three government-operated sub-district hospitals, called Upazila Health Complexes (UHCs), and the referral government hospital in Sylhet city (Osmani Medical College), all of which are staffed by physicians and nurses. The UHCs operate outpatient clinics for children under five and provide basic inpatient paediatric care, including oxygen. The majority of government provided inpatient care is provided at Osmani Medical College. Female CHWs employed by Projahnmo conduct bimonthly household surveillance, with a subset of CHWs providing weekly surveillance as a part of a PCV effectiveness study. Projahnmo CHWs conduct basic clinical assessments and refer ill children for care at the UHCs; they do not administer medicines themselves. Since 2015, a National Institutes of Health-funded study (K01TW009988) trained and supplied all Projahnmo clinical staff in Bangladesh, including CHWs, with pulse oximeters to screen children for hypoxemia, using the Masimo Rad5® oximeter and the LNCS® Y-I Multisite wrap probe (Figure 1b).

For peer review only

For peer review only - http://bmjopen.bmj.com/site/about/guidelines.xhtml

In Malawi, CHWs individually own the oximeters, and facilities were given a device for each clinic or ward, while in Bangladesh, Projahnmo owns the oximeters and individual healthcare providers are responsible for routine care and maintenance of the devices. Oximetry was not included in the Malawi paediatric guidelines, and Bangladesh did not have national paediatric pneumonia guidelines at the time of the study.

Design:

We conducted focus group discussions (FGDs). We planned three FGDs in each country, aiming to recruit between 6 and 10 people for each FGD (up to 60 participants in total). This number of groups was agreed upon before data collection began, driven by practical considerations given few healthcare workers in either setting have experience using pulse oximeters with children. The groups were planned to be CHWs, health centre or UHC staff, and referral hospital staff separately. Conducting separate FGDs for the different types of healthcare workers was to allow context-specific discussions and encourage participants with varying training backgrounds to feel confident about raising challenges relevant to their specific setting.

Sampling:

HCPs were purposefully sampled from sites where pulse oximetry had been introduced, and the participants had received some form of training or mentorship in oximetry. Participants were identified by local researchers (BZ in Malawi and SA in Bangladesh) to be a representative sample of HCPs from their setting (e.g. small and large health centres, inpatient wards and outpatient departments in the hospital), and contacted directly by phone. All HCPs contacted participated. Participants were reimbursed for their travel costs to the local healthcare facility and provided with refreshments.

Procedure:

FGDs were led by local researchers with experience in conducting qualitative research, with support from a facilitator with knowledge of pulse oximeters. The FGDs were divided into two sections, the first addressing the participants' personal experience with using pulse oximeters. The topic guide included: positive and negative experiences, and possible improvements and challenges (Web appendix 1). During the second part of the discussion, the participants were presented with different probe designs and given an opportunity to use them for an hour (Web appendix 2). Following this, the discussion addressed positive and negative aspects of the different designs to encourage critical thinking of possible design solutions to the current limitations of a universal paediatric probe.

For peer review only - http://bmjopen.bmj.com/site/about/guidelines.xhtml

For peer review only

The FGDs were audio recorded and then transcribed, along with the facilitators notes. Questions were asked in a mix of English and local dialects depending on understanding and ease of expression (Chichewa, Bangla or Sylheti) and participants were told to answer in their preferred language. Responses were clarified by facilitators if there was an issue with language and understanding between participants. Recordings were transcribed and translated where necessary. Translations for Malawi were done by BZ and EK together until final transcripts were agreed, and by an independent professional service for Bangladesh.

Analysis:

We analysed the data thematically using a framework approach, as an appropriate method for a multi-disciplinary team conducting health research [21]. This process involves five steps: familiarisation, identifying a thematic framework, indexing, mapping and interpretation [22]. The transcripts and notes from the FGDs were printed and coded on paper, with the coding matrix created in Microsoft Excel. CK and KF independently familiarised themselves and indexed the data, and the emergent themes were discussed until a consensus was reached on the mapping and interpretation of the data. This interpretation was shared with the local researchers (BZ and EK in Malawi; EDM and MI in Bangladesh) for further discussion until all were in agreement.

Ethics:

Written informed consent was obtained from all FGD participants. This study was reviewed and approved by the University College London research ethics committee (8075/003), Johns Hopkins Medicine Institutional Review Board (IRB00047406), the Malawi National Health Sciences Research Committee (16/4/1570) and Bangladesh Medical Research Council (BMRC/NREC/2013-2016/1272).

Results

We conducted six FGDs, with 23 participants from Bangladesh and 26 from Malawi (Table 1). We identified five emergent themes: trust; value; user-related experience; sustainability; and design.

Trust

Trust emerged as a theme both in terms of how the HCPs interpret the oximetry results, and how caregivers interact with HCPs and the pulse oximeter. We found that all cadres of HCPs in both sites had an awareness of the fallibility of the oximetry readings, specifically relating to lower SpO_2 values. For SpO_2 levels which were deemed abnormal, <90% up to <95% according to different participants,

For peer review only - http://bmjopen.bmj.com/site/about/guidelines.xhtml

HCPs stated that they would often re-check the result before making a referral or treatment decision:

"if we see it is 89% we change the probe or change the finger" (Hospital, Bangladesh)

However, questioning the validity of these lower SpO_2 results in the context of a child's clinical condition was only discussed by the HCPs who worked in the hospital setting. This difference in the trust placed in the SpO_2 results by different types of HCPs suggests that more in-depth clinical training and understanding of the technology may result in different clinical applications:

"*sometimes the pulse oximeter can give readings which you are not comfortable with according to the presentation of the child […] most of the time when it happens like that, we just use our judgement"* (Hospital, Malawi)

An outcome of using pulse oximeters for pneumonia diagnosis was a change in parental and community understanding and perceptions of care, with HCPs discussing increased trust in their referral and treatment decisions. This worked in two ways, firstly with the oximeter acting as a direct feedback and education tool:

"*if the mother is able to read you can show the exact figure and she will accept the treatment of oxygen, [before] it was very difficult to explain the role or the importance of the oxygen machine and some mothers refused*" (Hospital, Malawi)

Secondly, in Malawi HCPs projected that the oximeters had improved clinical care, and therefore outcomes, which led caregivers to be more inclined to accept the referral or treatment being recommended, especially in the case of oxygen:

"[previously] in the village they were saying that when a child is put on the oxygen machine it facilitates death, therefore it was making problems, but this time because children are put on oxygen earlier they survive, it's because we knew the saturation" (Health centre, Malawi)

Value

The theme of value relates to the inherent value of improved decision making, HCPs perceived self-value (i.e. confidence) in their clinical work, and the physical value placed on maintaining a working pulse oximeter. As pneumonia is classified using a range of non-specific and often subjective clinical signs, HCPs discussed the positive addition of this more objective measure:

"…by looking at this we can understand how much respiratory distress is in there. Of course this helps us a lot." (Health Centre, Bangladesh)

For peer review only - http://bmjopen.bmj.com/site/about/guidelines.xhtml

In both sites HCPs from frontline settings (CHWs, health centres and UHCs) stated that the pulse oximeters had changed the way they work and given them confidence in making referral decisions. Interestingly however, in the referral hospital setting in Bangladesh where staff training is higher, very little value was placed on the pulse oximeter for improving their clinical performance, with the ability to conduct chest x-rays, lung ultra-sound and their clinical judgement valued more highly:

"…its sensitivity and specificity is very negligible to be taken as a diagnostic tool." (Hospital, Bangladesh)

In Bangladesh the CHWs reported pride in using the oximeters. In Malawi, the CHWs placed a physical value on the oximeters and discussed the personal effort, such as paying out of pocket to travel commercial charging services, they put in to maintaining a working device:

"…we have been trying all that is humanly possible to take care of these things, but it only becomes a problem when it comes to the issue of charging." (CHW, Malawi)

This was also reflected at the health centre, where not all facilities have electricity and one or two staff are responsible for assessing children. At the referral hospital however this was not discussed, with oximeters belonging to the ward, which has a more consistent power supply. Ward-based ownership was discussed as a challenge, suggesting individual ownership could result in improved care and maintenance as having a device in working order would not be dependent on the performance of others.

"…some of the clinicians do not take care of them, so when the machine is not working it means the whole department is affected" (Hospital, Malawi)

<u>User-related experience</u>

HCPs at all levels discussed their experiences of using pulse oximeters in children under 5-years, presenting challenges, their solutions and perceptions of usability. The time taken to get a measurement ranged widely, with CHWs in Bangladesh agreeing measurements took less than 1 minute but in Malawi that it could take up to 20 minutes. The factors that increased the time taken to get a measurement were consistently cited as movement and physically smaller children, and in Malawi dirty toes making measurements difficult:

"Getting readings from irritable babies is a bit tough and it takes time." (Health centre, Bangladesh)

"…using it on a child up to six months of age, sometimes it has been a problem because these children have got small fingers, so although we use toes sometimes they are also small and the child

For peer review only - http://bmjopen.bmj.com/site/about/guidelines.xhtml

is afraid so they start crying. So we have got other things we can give a child to play with but it is a little bit of a problem, but at the end we get the results." (CHW, Malawi)

Solutions to these challenging children included asking caregivers to breastfeed, giving them a toy to distract them, and simply waiting. The term used frequently to describe challenging children was 'fear', with the HCPs stating that children are afraid of having the measurements taken. This fear was associated with the sensors' red light which frightened children, the anticipation of pain, or just being an unknown. All of these could result in the child being agitated, crying and uncooperative. Despite these issues in small and agitated infants, the oximeters were considered easy to use:

"…it's not complicated, it doesn't need complicated education for a healthcare worker to use, with a good explanation from a colleague or friend you are able to use it." (Hospital, Malawi)

There was also the acknowledgement that time to reading was not as important as getting the correct measurement; for some respondents, the reason some measurements take longer is the desire to get a reliable reading. This included cleaning the child's digits or repositioning the probe:

"…taking longer does not mean that one doesn't know the procedure, but sometime it's because you want to give the correct reading." (CHW, Malawi)

A key challenge reported by frontline HCPs in Malawi was around keeping the oximeter charged; this was not considered a significant challenge in Bangladesh. However, here they had issues with ensuring the oximeter remained dry and protected during rains and being fully waterproof was desirable. Depending on usage, battery life was reported as 1 week – 2 months.

<u>Sustainability</u>

Sustainability was discussed in terms of the device's durability, and the need for continued professional development. Generally the pulse oximeters were thought of as robust and durable, with some of the HCPs having used their device for over four years without replacements. However, the battery was highlighted as the least durable part of the device, and there was a perception that when the battery was worn down the readings became less reliable.

"There is a matter with the battery too, if the battery is not enough the reading takes a long time to appear. It sometimes gives false negative readings." (Hospital, Bangladesh)

This related to the HCPs suggestion of having on-going maintenance support rather than wanting replacement devices. HCPs described the need for on-going training and support, but also expressed

For peer review only - http://bmjopen.bmj.com/site/about/guidelines.xhtml

a desire for more in-depth education on how oximetry works which goes beyond the basic training to take a reliable measurement:

"A person gets used to what they are doing once they have been oriented. I think sometimes it's also good for you and your team to orient us on how this thing works [...] the way this thing works, we don't know" (Health centre, Malawi)

In terms of keeping the devices clean and properly stored, an important factor for prolonging shelf-life, we found conflicting opinions between Malawi and Bangladesh. Malawi deemed the probes easy to clean and store securely, although the light colour and materials of the device was thought to show dirt easily. However, in Bangladesh cleaning was described as burdensome; this likely reflects the different devices and therefore methods needed for cleaning, or different perceptions of the importance and frequency of cleaning.

"It is hard work to clean it with hexsol and cotton after coming back from the field every day. If we could get something else to clean it with so that we can clean once a week, I don't like cleaning it every day." (CHW, Bangladesh)

Design

The key challenges mentioned repeatedly across sites and HCP cadres were the battery, sensitivity of the probe in relation to movement or low perfusion, and the probe fit in younger children. Panel 1 summarises the design features requested or suggested to improve the pulse oximeters for use in these low-resource settings. Suggestions covered the probe, such as having detachable probes of different sizes, charging and battery life, such as additional power packs and solar charging, and features to help with agitated children.

The oximeters which HCPs used were designed for continuous monitoring; therefore oxygen saturation is not a single static result. This was seen as a negative, with HCPs in both sites wanting the ability to stop at a result and even store measurements (e.g. a blood glucose monitor):

"Readings would fluctuate if the baby moves. We don't want that. After getting the actual reading it should stay fixed." (CHW, Bangladesh)

In Bangladesh specifically, the CHWs stated a preference for numbers or bars to indicate measurement quality, rather than a dynamic waveform display. This opinion was not reflected in Malawi, which could be a result of using different devices or different training. A specific challenge presented by CHWs in Malawi was the use of the oximeter in direct sunlight; CHWs often hold clinics

For peer review only - http://bmjopen.bmj.com/site/about/guidelines.xhtml

outside and they faced the combined challenges of bright sunlight and dust, both of which they reported as challenges in taking measurements.

"…it returns the correct results when you are in the shade, but while you are in sunlight it fails to determine good results." (CHW, Malawi)

Positive design features included the portability of devices, the ease of using them and perceived durability, with little direct criticism of the oximeters that the HCPs had been using:

"…of the things I like most about using the pulse oximeter, the first one is the portability, because I can use it anywhere." (Hospital, Malawi)

Discussion

We investigated end-user experiences of using pulse oximeters by a range of different HCPs across clinical settings in Malawi and Bangladesh. The FGDs highlighted similarities in experience, such as challenges in battery durability, the difficulty of small and agitated children and the positive impact of oximeters on clinical practice. However, there were key differences between the providers' experiences in Malawi and Bangladesh and between HCP cadres.

Of note was the difference in perceived ease of cleaning, which was seen as more burdensome in Bangladesh. This is likely associated with the Y-shaped wrap probe design, compared to the more easily cleaned clip design used in Malawi (Figure 1). Interestingly though, most critiques were similar between sites, highlighting some of the major challenges of using pulse oximetry in children – namely movement, low perfusion and small digits. This consistency between our sampled HCPs from each site suggests these challenges are not device dependent and therefore a specifically designed re-usable device for universal paediatric use in low-resource settings is needed.

We identified differences in the sense of value placed on the oximeters by the HCPs, with the higher trained HCPs attributing less value to the results than the HCPs with more limited training. Those with more training valued their clinical judgement more and were more willing to question the accuracy of SpO_2 results. This poses interesting lessons for scaling-up implementation and training, as despite perceptions that obtaining a SpO_2 measurement is generally easy, the interpretation of the result is more nuanced. Sustained mentorship and more in-depth training were desired by the HCPs, and this needs to be considered as part of any implementation programme.

For peer review only - http://bmjopen.bmj.com/site/about/guidelines.xhtml

As the oximeters were used as spot-check devices rather than continuous monitors, as would generally be found in operating theatres or high-dependency care in high-income settings, many of the suggested design changes related to improving the devices for this process. One example of this was the need for improved battery-life and charging, with HCPs highlighting their limited ability to easily access charging points, unlike high-income inpatient settings. Consistently, the desire for quicker, static results and a movement tolerant probe with improved fit on younger infants was important. Unexpected issues, such as usability in direct sunlight, emphasize the importance of end-user engagement in product development as clinical devices designed for high-income settings would not need to be robust to outdoor use.

The idea of a pulse oximeter being able to improve trust between a caregiver and healthcare provider poses potentially exciting opportunities for improving referral and treatment for paediatric pneumonia. Early diagnosis and treatment as downstream in the health system as possible, ideally to the level of CHWs, are key strategies for improving pneumonia outcomes and therefore reducing morbidity and mortality burden [23]. Therefore, an objective and simple clinical tool with in-built decision support, e.g. auditory or visual alarms when the SpO_2 is outside of normal range, presents an opportunity for caregiver education and empowerment in the referral decision-making process. Recent data from Malawi supports the potential for oximetry to improve referral decision-making in frontline settings, with HCPs more than twice as likely to correctly refer children with a SpO_2 <90% compared to those with a $SpO_2 \geq 90\%$ when using an oximeter during routine outpatient care [12]. Interestingly, this has not necessarily been the case with other more objective diagnostic tools, with examples of rapid diagnostic malaria tests leading to provider-caregiver tensions around treatments [24 25].

This study was potentially subject to social-desirability bias, with healthcare workers expressing opinions which they thought the facilitators wanted to hear. The purpose of the study was explained to the participants during the consent process, and was highlighted as an opportunity for them to contribute to the design of a revised paediatric oximeter and probe. In addition, the groups in some cases were mixed in terms of gender and job titles, possibly influencing participant's confidence in expressing their views and experiences. To mitigate these potential biases, the facilitators encouraged the all participants to contribute to the discussions and to be critical throughout. Both positive and negative views were given in both Malawi and Bangladesh, and by different types of HCPs, therefore we do not feel these biases detract from our findings. Finally, we were limited by the number of groups we conducted; additional groups or a different sampling approach may have

For peer review only

For peer review only - http://bmjopen.bmj.com/site/about/guidelines.xhtml

led to alternative perspectives being included, as the number was not driven by saturation. Therefore the conclusions we draw need to be interpreted accordingly.

Overall pulse oximeters were valued by the HCPs we sampled for this study, despite challenges with charging, maintenance and speed of achieving accurate readings in moving or smaller children. This implies that making improvements to currently available oximeters and probes could further facilitate successful implementation of this technology at the community through to the hospital level for routine paediatric care in these two settings. Based on these providers varied experiences, we recommend that efforts to re-design a pulse oximeter for paediatric spot-checks focus on improvements to battery durability, better fit for smaller digits and the speed at which readings are obtained; these were all important challenges which did not necessarily have local solutions presented. More substantive design changes could focus on alternative power and charging systems (e.g. solar charging) and '3-in-1' devices which include respiratory rate and temperature measurements.

For peer review only - http://bmjopen.bmj.com/site/about/guidelines.xhtml

Acknowledgments

We would like to thank all those healthcare providers who took part in the focus group discussions for their time. We would like to acknowledge other members of the study team: Katie Fernandez (Lifebox); Charles Makwenda and Tambosi Phiri (Parent and Child Health Initiative); Tim Colbourn (University College London); Mike Bernstein (Physio Monitor); and Nazma Begum, Arun Dutta Roy and Abu Abdullah Mohammad Hanif (Johns Hopkins University-Bangladesh).

Funding

This study was funded by the Bill & Melinda Gates Foundation (grant number: OPP1133291).

Conflict of Interest

Authors declare no conflicts of interest.

Author contributions

The qualitative study was designed and topic guides developed by IWi, IWa, CK and EDM, and the field manual written by CK. Oversight of the study was conducted by CK, BN and BZ in Malawi and EDM, AB and MI in Bangladesh. In Malawi, BZ and EK arranged, conducted, transcribed and translated the FGDs. In Bangladesh SA and MI arranged and conducted the FGDs. The data was coded and analysed by CK. The manuscript was written by CK, with considerable input from EDM. IWi, IWa, EDM, BZ, EK, SA, MI, NB, AB and BN read, commented and approved the manuscript.

Data Sharing Statement

Anonymised transcripts can be shared, following the signing of a data sharing agreement, subject to approval from the relevant National ethics committees. For further information please contact Dr. Carina King: c.king@ucl.ac.uk.

For peer review only

References

1. Sazawal S, Black RE, Pneumonia Case Management Trials G. Effect of pneumonia case management on mortality in neonates, infants, and preschool children: a meta-analysis of community-based trials. Lancet Infect Dis 2003;**3**(9):547-56.

2. Niessen LW, ten Hove A, Hilderink H, et al. Comparative impact assessment of child pneumonia interventions. Bull World Health Organ 2009;**87**(6):472-80.

3. Walker CL, Rudan I, Liu L, et al. Global burden of childhood pneumonia and diarrhoea. Lancet 2013;**381**(9875):1405-16.

4. Organisation WH. *Oxygen therapy for children: a manual for health workers*. Geneva: WHO, 2016.

5. . WHO Recommendations on the Management of Diarrhoea and Pneumonia in HIV-Infected Infants and Children: Integrated Management of Childhood Illness (IMCI). Geneva, 2010.

6. Orimadegun A, Ogunbosi B, Orimadegun B. Hypoxemia predicts death from severe falciparum malaria among children under 5 years of age in Nigeria: the need for pulse oximetry in case management. Afr Health Sci 2014;**14**(2):397-407.

7. Chhibber AV, Hill PC, Jafali J, et al. Child Mortality after Discharge from a Health Facility following Suspected Pneumonia, Meningitis or Septicaemia in Rural Gambia: A Cohort Study. Plos One 2015;**10**(9):e0137095.

8. Hooli S, Colbourn T, Lufesi N, et al. Predicting Hospitalised Paediatric Pneumonia Mortality Risk: An External Validation of RISC and mRISC, and Local Tool Development (RISC-Malawi) from Malawi. Plos One 2016;**11**(12):e0168126.

9. Wandeler G, Pauchard JY, Zangger E, et al. Which clinical signs predict hypoxaemia in young Senegalese children with acute lower respiratory tract disease? Paediatrics and International Child Health 2015;**35**(1):65-68.

10. Basnet S, Adhikari RK, Gurung CK. Hypoxemia in children with pneumonia and its clinical predictors. The Indian Journal of Pediatrics 2006;**73**(9):777-81.

11. Lodha R, Bhadauria PS, Kuttikat AV, et al. Can Clinical Symptoms or Signs Accurately Predict Hypoxemia in Children with Acute Lower Respiratory Tract Infections? Indian Pediatr 2003;**41**:129-35.

12. McCollum ED, King C, Deula R, et al. Pulse oximetry for children with pneumonia treated as outpatients in rural Malawi. Bull World Health Organ 2016;**94**:893-902.

13. Duke T, Subhi R, Peel D, et al. Pulse oximetry: technology to reduce child mortality in developing countries. Ann Trop Paediatr 2009;**29**(3):165-75.

For peer review only - http://bmjopen.bmj.com/site/about/guidelines.xhtml

14. Emdin CA, Mir F, Sultana S, et al. Utility and feasibility of integrating pulse oximetry into the routine assessment of young infants at primary care clinics in Karachi, Pakistan: a cross-sectional study. BMC Pediatrics 2015;**15**(1):141.

15. King EM, Lieu C, Kasasa A, et al. Pulse Oximetry as a Screening Tool to Detect Hypoxia Associated with Early-onset Sepsis in Asymptomatic Newborns: A Feasibility Study in a Low-income Country. British Journal of Medicine & Medical Research 2014;**4**(5):1115-28.

16. Ginsburg AS, Izadnegahdar R, Klugman KP. World Pneumonia Day 2016: pulse oximetry and oxygen. The Lancet Global Health 2016;**4**(12):e893-e94.

17. Ginsburg AS, Van Cleve WC, Thompson MI, et al. Oxygen and pulse oximetry in childhood pneumonia: a survey of healthcare providers in resource-limited settings. J Trop Pediatr 2012;**58**(5):389-93.

18. Spence H, Baker K, Wharton-Smith A, et al. Childhood pneumonia diagnostics: community health workers' and national stakeholders' differing perspectives of new and existing aids. Glob Health Action 2017;**10**(1):1290340.

19. Nsona H, Mtimuni A, Daelmans B, et al. Scaling up Integrated Community Case Management of Childhood Illness: Update from Malawi. Am J Trop Med Hyg 2012;**87**(Suppl 5):54-60.

20. Malalwi Go. Integrated Management of Childhood Illness - Caring for Newborns and Children in the Community: Manual for Healt hSurveillance Assistants. In: Health Mo, ed. Lilongwe, Malawi, 2008.

21. Gale NK, Heath G, Cameron E, et al. Using the framework method for the analysis of qualitative data in multi-disciplinary health research. BMC Medical Research Methodology 2013;**13**(1):117.

22. Ritchie J, Spencer L. Qualitative data analysis for applied policy research. The qualitative researcher's companion 2002;**573**:305-29.

23. McCollum ED, King C, Hammitt LL, et al. Reduction of childhood pneumonia mortality in the Sustainable Development era. Lancet Respir Med 2016;**4**(12):932-33.

24. Ansah EK, Reynolds J, Akanpigbiam S, et al. "Even if the test result is negative, they should be able to tell us what is wrong with us": a qualitative study of patient expectations of rapid diagnostic tests for malaria. Malaria Journal 2013;**12**(1):258.

25. Hutchinson E, Reyburn H, Hamlyn E, et al. Bringing the state into the clinic? Incorporating the rapid diagnostic test for malaria into routine practice in Tanzanian primary healthcare facilities. Glob Public Health 2015:1-15.

For peer review only - http://bmjopen.bmj.com/site/about/guidelines.xhtml

**Table 1:** Summary of the FGD participants and their work experience			
	Community level	Health centre or Upazila Health Complex	Hospital
**BANGLADESH**			
Total participants	8	7	8
Job titles (number)	Community healthcare worker (8)	Physician (4)<br>Medical officer (3)	Senior staff nurse (1)<br>Associate professor (2)<br>ICU staff (1)<br>Anaesthesiologist (1)<br>Assistant registrar (1)<br>Intern medical officer (1)
Work experience (mean, range)	1.7 years (0.6 – 4)	2.3 years (1 – 6)	14.7 years (0.5 – 32)
**MALAWI**			
Total participants	9	8	9
Job titles	Community healthcare worker (8)<br>Vital signs assistant (1)	Medical assistant (7)<br>Medical technician (1)	Clinical officer (3)<br>Nurse midwife (3)<br>Medical assistant (3)
Years' work experience (mean)	10.6 years (5 – 20)	8.3 years (3 – 23)	8.1 years (4 – 13)

For peer review only - http://bmjopen.bmj.com/site/about/guidelines.xhtml

Panel 1: Suggestions of desirable features or improvements given by healthcare providers

**Challenge:**	**Design suggestion:**
Probe fit	Supplied with multiple sizes of probes for different ages<br>A single cable with multiple probes that can be changed (e.g. clipped into the cable)<br>Softer material for a more comfortable fit
Probe placement	Probe made of transparent material so sensor placement on the nail can be seen
Cleaning	Alcohol wipes provided for easier cleaning<br>Different colour probe to make it easier to see the dirt, but does not look dirty quickly
Power	Solar powered charger with rechargeable batteries<br>Back-up power bank<br>Supplied with a spare battery
Agitated children	Toy feature in the device to distract the child<br>Improve the sensitivity of the device to be quicker<br>Improve the sensitivity of the device to tolerate movement
Integrated spot-check device	Store results in a memory that can later be accessed<br>Static oxygen saturation result display<br>'3-in-1' device which includes temperature and respiratory rate measurements as well<br>Shorter cable length for easier portability

For peer review only

For peer review only - http://bmjopen.bmj.com/site/about/guidelines.xhtml

Figure legends:

Figure 1: Pulse oximeters and probe used by healthcare providers in routine clinical care

a. Lifebox® oximeter and adult universal clip probe used in Malawi (accessed on 1st July 2017 from: www.lifebox.org)
b. Masimo Rad5® oximeter and LNCS® Y-I Multisite wrap probe used in Bangladesh (accessed on 1st July 2017 from: www.pacificmedicalsupply.com)

For peer review only

For peer review only - http://bmjopen.bmj.com/site/about/guidelines.xhtml

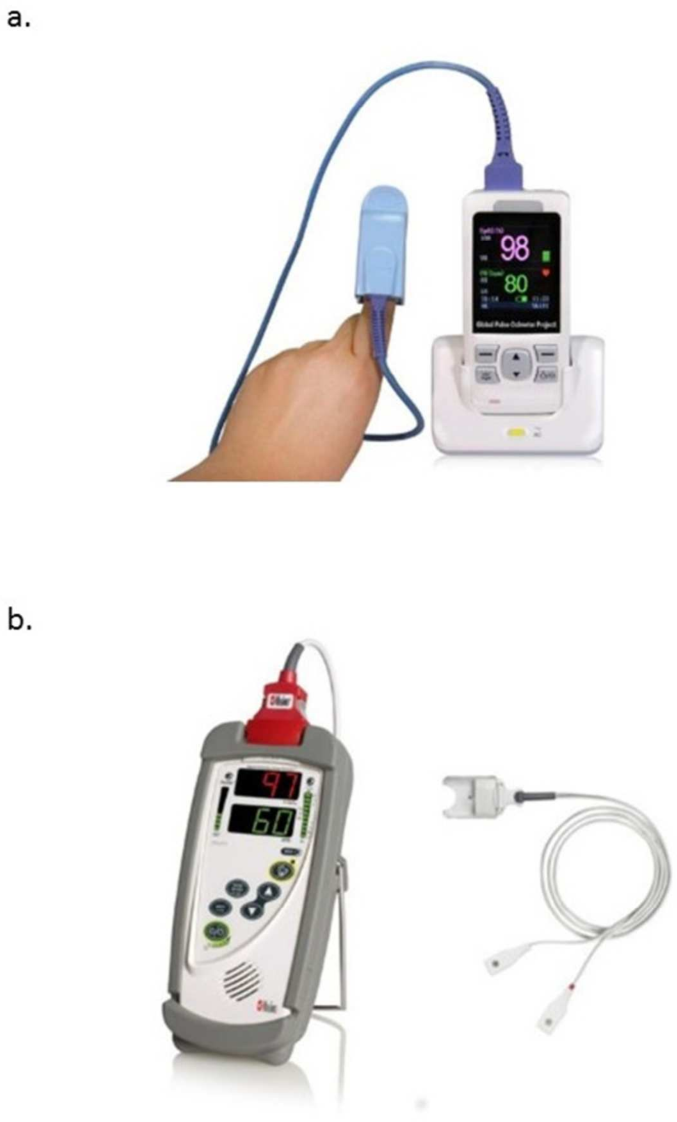

64x97mm (300 x 300 DPI)

Web Appendix 1 – Topic Guide

- What is your experience of using pulse oximeters in children?
- What have been the main issues you've encountered when using pulse oximeters?
- What have been the things you like most about using the pulse oximeters?
- What type of probes have you used? Have any been better than others? Why?
- Thinking about the probes, we would like to hear your feedback about some aspects of using them: ease of putting and keeping the probe on the child, durability, ease of taking a reading, ease of keeping it clean and storage
- Thinking about the oximeter, we would like to hear your feedback about some aspects of using them: ease of reading the display, durability, battery life and charging, time taken to get a reading
- What things would make the probe and pulse oximeter easier to use?
- What things would make the probe and pulse oximeter harder to use?

For peer review only

Appendix 2: Summary of pulse oximeter probes presented during focus group discussions with healthcare providers

Probe type	Figure	Product code
Neonatal wrap	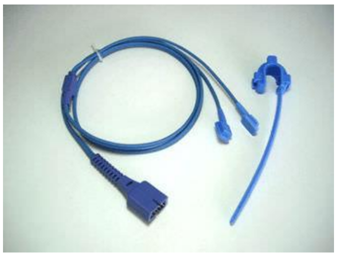	Acare ASYNR-D1
Adult clip	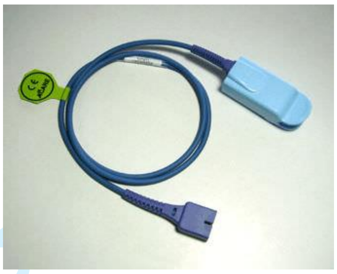	Acare ASANR-D1
Paediatric clip	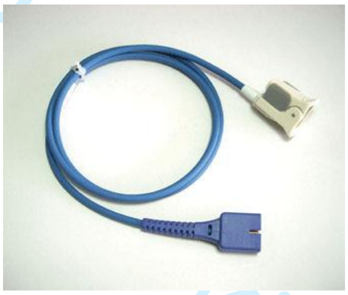	Acare ASPNR-D1
Ear clip	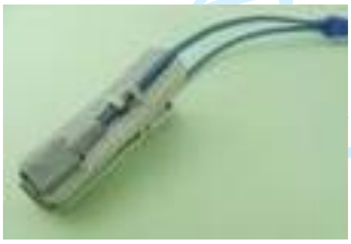	Nellcor U401-2HL
Adult boot	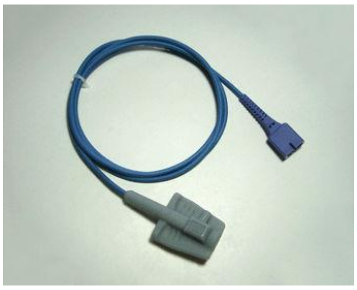	Acare ASSNR-D1
Paediatric boot	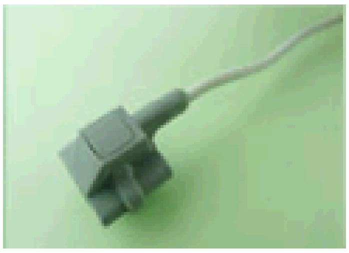	Nellcor U401-2EL

For peer review only

COREQ Checklist

No	Item	Guide questions/description	Page / evidence
Domain 1: Research team and reflexivity			
Personal Characteristics			
1.	Interviewer/ facilitator	Which author/s conducted the interview or focus group?	Pg 15, Author contributions
2.	Credentials	What were the researcher's credentials? *E.g. PhD, MD*	A mix of diploma, BSc, MSc and PhD
3.	Occupation	What was their occupation at the time of the study?	Pg 6, Methods: procedure
4.	Gender	Was the researcher male or female?	Both
5.	Experience and training	What experience or training did the researcher have?	Pg 6, 'Methods: Procedure'
Relationship with participants			
6.	Relationship established	Was a relationship established prior to study commencement?	No
7.	Participant knowledge of the interviewer	What did the participants know about the researcher? e.*g. personal goals, reasons for doing the research*	Pg 15, Discussion
8.	Interviewer characteristics	What characteristics were reported about the interviewer/facilitator? e.g. *Bias, assumptions, reasons and interests in the research topic*	Pg 15, Discussion
Domain 2: study design			
Theoretical framework			

For peer review only

No	Item	Guide questions/description	Page / evidence
9.	Methodological orientation and Theory	What methodological orientation was stated to underpin the study? *e.g. grounded theory, discourse analysis, ethnography, phenomenology, content analysis*	Pg 7, Methods: Analysis
Participant selection			
10.	Sampling	How were participants selected? *e.g. purposive, convenience, consecutive, snowball*	Pg 6, Methods: Sampling
11.	Method of approach	How were participants approached? e.*g. face-to-face, telephone, mail, email*	Pg 6, Sampling
12.	Sample size	How many participants were in the study?	Pg 7, Results and Table 1
13.	Non-participation	How many people refused to participate or dropped out? Reasons?	Pg 6, Sampling
Setting			
14.	Setting of data collection	Where was the data collected? e.*g. home, clinic, workplace*	Pg 6, Sampling
15.	Presence of non-participants	Was anyone else present besides the participants and researchers?	Pg 6, Methods: Procedure
16.	Description of sample	What are the important characteristics of the sample? *e.g. demographic data, date*	Table 1
Data collection			
17.	Interview guide	Were questions, prompts, guides provided by the authors? Was it pilot tested?	Appendix 1
18.	Repeat interviews	Were repeat interviews carried out? If yes, how many?	Not applicable

For peer review only

No	Item	Guide questions/description	Page / evidence
19.	Audio/visual recording	Did the research use audio or visual recording to collect the data?	Pg 7, Methods: Procedure
20.	Field notes	Were field notes made during and/or after the interview or focus group?	Pg 7, Methods: Procedure
21.	Duration	What was the duration of the interviews or focus group?	Between 1 and 2 hours
22.	Data saturation	Was data saturation discussed?	Pg 14, Discussion
23.	Transcripts returned	Were transcripts returned to participants for comment and/or correction?	This was not possible due to language potential barriers
Domain 3: analysis and findings			
Data analysis			
24.	Number of data coders	How many data coders coded the data?	Pg 7, Methods: Analysis
25.	Description of the coding tree	Did authors provide a description of the coding tree?	Pg 7, Methods: Analysis
26.	Derivation of themes	Were themes identified in advance or derived from the data?	Pg 7, Results
27.	Software	What software, if applicable, was used to manage the data?	Pg 7, Methods: Analysis
28.	Participant checking	Did participants provide feedback on the findings?	No, but discussed with local researchers (pg 7)

For peer review only

No	Item	Guide questions/description	Page / evidence
Reporting			
29.	Quotations presented	Were participant quotations presented to illustrate the themes / findings? Was each quotation identified? e.*g. participant number*	Throughout results section (pg 7 – 12)
30.	Data and findings consistent	Was there consistency between the data presented and the findings?	Throughout results section (pg 7 – 12)
31.	Clarity of major themes	Were major themes clearly presented in the findings?	Pg 7, Results
32.	Clarity of minor themes	Is there a description of diverse cases or discussion of minor themes?	Throughout results section (pg 7 – 12)

For peer review only

BMJ Open

Opportunities and barriers in paediatric pulse oximetry for pneumonia in low-resource clinical settings: a qualitative evaluation from Malawi and Bangladesh

Journal:	*BMJ Open*
Manuscript ID	bmjopen-2017-019177.R2
Article Type:	Research
Date Submitted by the Author:	24-Nov-2017
Complete List of Authors:	King, C.; University College London Institute for Global Health,<br>Boyd, Nicholas; Great Ormond Street Hospital For Children NHS Trust, UCL Institute of Child Health<br>Walker, Isabeau; Great Ormond Street Hospital For Children NHS Trust, UCL Institute of Child Health<br>Zadutsa, Beatiwel; Parent and Child Health Initiative<br>Baqui, Abdullah; Johns Hopkins University Bloomberg School of Public Health, International Center for Maternal and Newborn Health<br>Ahmed, Salahuddin; Johns Hopkins University Bloomberg School of Public Health, International Center for Maternal and Newborn Health<br>Islam, Mazharul; Shahjalal University of Science and Technology, Department of Anthropology<br>Kainja, Esther; Parent and Child Health Initiative<br>Nambiar, Bejoy; Institute for Global Health<br>Wilson, Iain; Lifebox Foundation<br>McCollum, Eric ; Johns Hopkins School of Medicine, Eudowood Division of Paediatric Respiratory Diseases; Johns Hopkins University Bloomberg School of Public Health, Department of International Health
**Primary Subject Heading**:	Global health
Secondary Subject Heading:	Paediatrics, Respiratory medicine, Qualitative research, Diagnostics
Keywords:	Pulse oximeter, Oxygen saturation, Pneumonia, Children, Sub-Saharan Africa, South Asia

SCHOLARONE™ Manuscripts

Title: Opportunities and barriers in paediatric pulse oximetry for pneumonia in low-resource clinical settings: a qualitative evaluation from Malawi and Bangladesh

Authors: Carina King[1*], Nicholas Boyd[2], Isabeau Walker[2], Beatiwel Zadutsa[3], Abdullah H. Baqui[4], Salahuddin Ahmed[4], Mazharul Islam[5], Esther Kainja[3], Bejoy Nambiar[1], Iain Wilson[6], Eric D. McCollum[7,8]

Affiliations:

1. Institute for Global Health, University College London, London, UK
2. Great Ormond Street Hospital NHS Foundation Trust, UCL Institute of Child Health, London, UK
3. Parent and Child Health Initiative, Lilongwe, Malawi
4. International Center for Maternal and Newborn Health, Johns Hopkins Bloomberg School of Public Health, Baltimore, USA
5. Department of Anthropology, Shahjalal University of Science and Technology, Sylhet, Bangladesh
6. Lifebox Foundation, London, UK
7. Eudowood Division of Pediatric Respiratory Diseases, Johns Hopkins School of Medicine, Baltimore, USA
8. Department of International Health, Johns Hopkins Bloomberg School of Public Health, Baltimore, USA

***Corresponding author:**

Dr. Carina King; Email: c.king@ucl.ac.uk; Address: Institute for Global Health, 30 Guilford Street, London, WC1N 1EH; Telephone: +44 (0) 2076797619

Keywords: Pulse oximeter, oxygen saturation, pneumonia, sub-Saharan Africa, South Asia, children

Word Count: 4,111

For peer review only

For peer review only - http://bmjopen.bmj.com/site/about/guidelines.xhtml

Abstract

Objective: To gain an understanding of what challenges pulse oximetry for paediatric pneumonia management poses, how it has changed service provision and what would improve this device for use across paediatric clinical settings in low-income countries.

Design: Focus group discussions (FGDs), with purposive sampling and thematic analysis using a framework approach.

Setting: Community, front line outpatient and hospital outpatient and inpatient settings in Malawi and Bangladesh, which provide paediatric pneumonia care.

Participants: Healthcare providers (HCPs) from Malawi and Bangladesh who had received training in pulse oximetry and had been using oximeters in routine paediatric care, including community healthcare workers, non-physician clinicians or medical assistants, and hospital-based nurses and doctors.

Results: We conducted six FGDs, with 23 participants from Bangladesh and 26 from Malawi. We identified five emergent themes: trust; value; user-related experience; sustainability; and design. HCPs discussed the confidence gained through using oximeters, resulting in improved trust from caregivers and valuing the device; although there were conflicts between the weight given to clinical judgement versus oximeter results. HCPs reported the ease of using oximeters, but identified movement and physically smaller children as measurement challenges. Challenges in sustainability related to battery durability and replacement parts were reported, however many HCPs had used the same device longer than four years demonstrating robustness within these settings. Desirable features included back-up power banks and integrated respiratory rate and thermometer capability.

Conclusions: Pulse oximetry was generally deemed valuable by HCPs for use as a spot-check device in a range of paediatric low-income clinical settings. Areas highlighted as challenges by HCPs, and therefore opportunities for re-design, included battery charging and durability, probe fit and sensitivity in paediatric populations.

For peer review only - http://bmjopen.bmj.com/site/about/guidelines.xhtml

Strengths and Limitations

- This is the first study to report on end-user perceptions of opportunities, challenges and desirable design features of pulse oximeters used for paediatric pneumonia management in low-resource settings, including community and outpatient providers.
- A key strength was the wide range of healthcare provider perspectives included, from community to referral hospital settings in South Asia and sub-Saharan Africa.
- The study was limited to participant's experience of using specific pulse oximeters and therefore may lack generalizability to other paediatric pulse oximeters not used in these settings.

For peer review only

For peer review only - http://bmjopen.bmj.com/site/about/guidelines.xhtml

Introduction

Several interventions, such as Pneumococcal conjugate vaccine (PCV) and standardised guidelines for diagnosis and treatment, have led to reductions in pneumonia morbidity and mortality over the last twenty years [1 2]. However, in spite of these gains, pneumonia remains the leading cause of infectious mortality amongst children globally, with the vast majority of the burden falling in sub-Saharan Africa and south Asia [3]. To accelerate reductions in pneumonia mortality, further refinement of diagnosis and treatment pathways are needed, including correct referral and access to oxygen treatment [4].

Pulse oximetry non-invasively measures peripheral arterial oxyhemoglobin saturation (SpO_2). Hypoxemia (defined as an SpO_2 <90%) is included in the World Health Organization (WHO) guidelines as a pneumonia danger sign [5], and is associated with increased mortality from pneumonia, as well as other illnesses like malaria [6-8]. Recent evidence from Malawi has also indicated that a SpO_2 90-92% is predictive of mortality amongst children hospitalized with pneumonia [8].

While some studies have attempted to predict hypoxemia in children with pneumonia using a combination of clinical signs, there has been mixed success [9-11]. Clinical signs alone fail to identify a proportion of hypoxemic children based on the current WHO guidelines, which results in a missed opportunity for referral and appropriate treatment [12 13]. In addition, the subjectivity of clinical signs can lead to variation in care – especially among community healthcare workers (CHWs), who often lack ongoing supervision.

Pulse oximeters have been successfully used in low-resource paediatric settings, but are yet to be widely adopted, particularly during outpatient care [14 15]. The Ethiopian Ministry of Health has demonstrated leadership in this area, setting up an initiative in 2016 to ensure oximetry and oxygen therapy are available nationally across the healthcare system [16]. However, Ethiopia is an exception, with implementation of oximetry in other developing countries continuing to be slow. Implementation barriers cited include cost, issues with training and supervision and the lack of appropriately designed, robust oximeters and universal paediatric probes, although there are several initiatives to develop devices for low-income settings (e.g. Lifebox® and the Phone Oximeter™)[17-19].

In order to better understand current barriers to use of pulse oximetry by healthcare providers (HCPs) in a range of healthcare settings, and explore opportunities that this technology provides, input from end-users is needed [20]. With the ultimate goal of designing a universal paediatric probe

For peer review only - http://bmjopen.bmj.com/site/about/guidelines.xhtml

for all levels of healthcare services in resource-poor settings, we aimed to gain an understanding of the challenges of pulse oximetry, how its use has changed service provision and how current devices could be improved for these settings. This end-user perspective is currently limited in the literature and is essential to ensure investment in pulse oximetry is sustainable and effective.

Methods

We conducted a qualitative study with HCPs from different levels of the healthcare system in from one site in Malawi (Mchinji district, central region) and one in Bangladesh (Sylhet district, northeast region) from May – July 2016, as part of a wider programme of work aiming to design a universal paediatric oximeter probe.

Setting:

In Malawi there are three levels of government provided healthcare: CHWs (locally known as Health Surveillance Assistants), health centres and district hospitals. CHWs conduct weekly or bi-weekly village clinics and home visits providing basic integrated community case management (iCCM) for paediatric infections [21 22]. Health centres are outpatient facilities run by nurses, clinical officers or medical assistants, and District Hospitals have inpatient facilities with capacity for oxygen treatment. In Mchinji, pulse oximetry was successfully introduced into all three healthcare settings in 2012 as part of a PCV research project, using the Acare Technology AH-MX manufactured Lifebox® oximeter and universal adult clip probe (Figure 1a) [12].

In Bangladesh, the study was conducted at Projahnmo, a research consortium comprised of Johns Hopkins University and several local non-governmental organizations in partnership with the Bangladesh Ministry of Family Health and Welfare. Current Projahnmo activities are integrated within three government-operated sub-district hospitals, called Upazila Health Complexes (UHCs), and the referral government hospital in Sylhet city (Osmani Medical College), all of which are staffed by physicians and nurses. The UHCs operate outpatient clinics for children under five and provide basic inpatient paediatric care, including oxygen. The majority of government provided inpatient care is provided at Osmani Medical College. Female CHWs employed by Projahnmo conduct bimonthly household surveillance, with a subset of CHWs providing weekly surveillance as a part of a PCV effectiveness study. Projahnmo CHWs conduct basic clinical assessments and refer ill children for care at the UHCs; they do not administer medicines themselves. Since 2015, a National Institutes of Health-funded study (K01TW009988) trained and supplied all Projahnmo clinical staff in

For peer review only - http://bmjopen.bmj.com/site/about/guidelines.xhtml

Bangladesh, including CHWs, with pulse oximeters to screen children for hypoxemia, using the Masimo Rad5® oximeter and the LNCS® Y-I Multisite wrap probe (Figure 1b).

In Malawi, CHWs individually own the oximeters, and facilities were given a device for each clinic or ward, while in Bangladesh, Projahnmo owns the oximeters and individual healthcare providers are responsible for routine care and maintenance of the devices. Oximetry was not included in the Malawi paediatric guidelines, and Bangladesh did not have national paediatric pneumonia guidelines at the time of the study.

Design:

We conducted focus group discussions (FGDs). We planned three FGDs in each country, aiming to recruit between 6 and 10 people for each FGD (up to 60 participants in total). This number of groups was agreed upon before data collection began, driven by practical considerations given few healthcare workers in either setting have experience using pulse oximeters with children. The groups were planned to be CHWs, health centre or UHC staff, and referral hospital staff separately. Conducting separate FGDs for the different types of healthcare workers was to allow context-specific discussions and encourage participants with varying training backgrounds to feel confident about raising challenges relevant to their specific setting.

Sampling:

HCPs were purposefully sampled from sites where pulse oximetry had been introduced, and the participants had received some form of training or mentorship in oximetry. Participants were identified by local researchers (BZ in Malawi and SA in Bangladesh) to be a representative sample of HCPs from their setting (e.g. small and large health centres, inpatient wards and outpatient departments in the hospital), and contacted directly by phone. All HCPs contacted participated. Participants were reimbursed for their travel costs to the local healthcare facility and provided with refreshments.

Procedure:

FGDs were led by local researchers with experience in conducting qualitative research, with support from a facilitator with knowledge of pulse oximeters. The FGDs were divided into two sections, the first addressing the participants' personal experience with using pulse oximeters. The topic guide included: positive and negative experiences, and possible improvements and challenges (Web appendix 1). During the second part of the discussion, the participants were presented with different probe designs and given an opportunity to use them for an hour (Web appendix 2). Following this,

For peer review only - http://bmjopen.bmj.com/site/about/guidelines.xhtml

the discussion addressed positive and negative aspects of the different designs to encourage critical thinking of possible design solutions to the current limitations of a universal paediatric probe.

The FGDs were audio recorded and then transcribed, along with the facilitators notes. Questions were asked in a mix of English and local dialects depending on understanding and ease of expression (Chichewa, Bangla or Sylheti) and participants were told to answer in their preferred language. Responses were clarified by facilitators if there was an issue with language and understanding between participants. Recordings were transcribed and translated where necessary. Translations for Malawi were done by BZ and EK together until final transcripts were agreed, and by an independent professional service for Bangladesh.

Analysis:

We analysed the data thematically using a framework approach, as an appropriate method for a multi-disciplinary team conducting health research [23]. This process involves five steps: familiarisation, identifying a thematic framework, indexing, mapping and interpretation [24]. The transcripts and notes from the FGDs were printed and coded on paper, with the coding matrix created in Microsoft Excel. CK and KF independently familiarised themselves and indexed the data, and the emergent themes were discussed until a consensus was reached on the mapping and interpretation of the data. This interpretation was shared with the local researchers (BZ and EK in Malawi; EDM and MI in Bangladesh) for further discussion until all were in agreement.

Ethics:

Written informed consent was obtained from all FGD participants. This study was reviewed and approved by the University College London research ethics committee (8075/003), Johns Hopkins Medicine Institutional Review Board (IRB00047406), the Malawi National Health Sciences Research Committee (16/4/1570) and Bangladesh Medical Research Council (BMRC/NREC/2013-2016/1272).

Results

We conducted six FGDs, with 23 participants from Bangladesh and 26 from Malawi (Table 1). We identified five emergent themes: trust; value; user-related experience; sustainability; and design.

Trust

Trust emerged as a theme both in terms of how the HCPs interpret the oximetry results, and how caregivers interact with HCPs and the pulse oximeter. We found that all cadres of HCPs in both sites

For peer review only - http://bmjopen.bmj.com/site/about/guidelines.xhtml

had an awareness of the fallibility of the oximetry readings, specifically relating to lower SpO_2 values. For SpO_2 levels which were deemed abnormal, <90% up to <95% according to different participants, HCPs stated that they would often re-check the result before making a referral or treatment decision:

"if we see it is 89% we change the probe or change the finger" (Hospital, Bangladesh)

However, questioning the validity of these lower SpO_2 results in the context of a child's clinical condition was only discussed by the HCPs who worked in the hospital setting. This difference in the trust placed in the SpO_2 results by different types of HCPs suggests that more in-depth clinical training and understanding of the technology may result in different clinical applications:

"*sometimes the pulse oximeter can give readings which you are not comfortable with according to the presentation of the child […] most of the time when it happens like that, we just use our judgement"* (Hospital, Malawi)

An outcome of using pulse oximeters for pneumonia diagnosis was a change in parental and community understanding and perceptions of care, with HCPs discussing increased trust in their referral and treatment decisions. This worked in two ways, firstly with the oximeter acting as a direct feedback and education tool:

"*if the mother is able to read you can show the exact figure and she will accept the treatment of oxygen, [before] it was very difficult to explain the role or the importance of the oxygen machine and some mothers refused*" (Hospital, Malawi)

Secondly, in Malawi HCPs projected that the oximeters had improved clinical care, and therefore outcomes, which led caregivers to be more inclined to accept the referral or treatment being recommended, especially in the case of oxygen:

"[previously] in the village they were saying that when a child is put on the oxygen machine it facilitates death, therefore it was making problems, but this time because children are put on oxygen earlier they survive, it's because we knew the saturation" (Health centre, Malawi)

<u>Value</u>

The theme of value relates to the inherent value of improved decision making, HCPs perceived self-value (i.e. confidence) in their clinical work, and the physical value placed on maintaining a working pulse oximeter. As pneumonia is classified using a range of non-specific and often subjective clinical signs, HCPs discussed the positive addition of this more objective measure:

For peer review only

For peer review only - http://bmjopen.bmj.com/site/about/guidelines.xhtml

"…by looking at this we can understand how much respiratory distress is in there. Of course this helps us a lot." (Health Centre, Bangladesh)

In both sites HCPs from frontline settings (CHWs, health centres and UHCs) stated that the pulse oximeters had changed the way they work and given them confidence in making referral decisions. Interestingly however, in the referral hospital setting in Bangladesh where staff training is higher, very little value was placed on the pulse oximeter for improving their clinical performance, with the ability to conduct chest x-rays, lung ultra-sound and their clinical judgement valued more highly:

"…its sensitivity and specificity is very negligible to be taken as a diagnostic tool." (Hospital, Bangladesh)

In Bangladesh the CHWs reported pride in using the oximeters. In Malawi, the CHWs placed a physical value on the oximeters and discussed the personal effort, such as paying out of pocket to travel commercial charging services, they put in to maintaining a working device:

"*…we have been trying all that is humanly possible to take care of these things, but it only becomes a problem when it comes to the issue of charging." (CHW, Malawi)*

This was also reflected at the health centre, where not all facilities have electricity and one or two staff are responsible for assessing children. At the referral hospital however this was not discussed, with oximeters belonging to the ward, which has a more consistent power supply. Ward-based ownership was discussed as a challenge, suggesting individual ownership could result in improved care and maintenance as having a device in working order would not be dependent on the performance of others.

"*…some of the clinicians do not take care of them, so when the machine is not working it means the whole department is affected*" (Hospital, Malawi)

User-related experience

HCPs at all levels discussed their experiences of using pulse oximeters in children under 5-years, presenting challenges, their solutions and perceptions of usability. The time taken to get a measurement ranged widely, with CHWs in Bangladesh agreeing measurements took less than 1 minute but in Malawi that it could take up to 20 minutes. The factors that increased the time taken to get a measurement were consistently cited as movement and physically smaller children, and in Malawi dirty toes making measurements difficult:

"Getting readings from irritable babies is a bit tough and it takes time." (Health centre, Bangladesh)

For peer review only - http://bmjopen.bmj.com/site/about/guidelines.xhtml

"…using it on a child up to six months of age, sometimes it has been a problem because these children have got small fingers, so although we use toes sometimes they are also small and the child is afraid so they start crying. So we have got other things we can give a child to play with but it is a little bit of a problem, but at the end we get the results." (CHW, Malawi)

Solutions to these challenging children included asking caregivers to breastfeed, giving them a toy to distract them, and simply waiting. The term used frequently to describe challenging children was 'fear', with the HCPs stating that children are afraid of having the measurements taken. This fear was associated with the sensors' red light which frightened children, the anticipation of pain, or just being an unknown. All of these could result in the child being agitated, crying and uncooperative. Despite these issues in small and agitated infants, the oximeters were considered easy to use:

"…it's not complicated, it doesn't need complicated education for a healthcare worker to use, with a good explanation from a colleague or friend you are able to use it." (Hospital, Malawi)

There was also the acknowledgement that time to reading was not as important as getting the correct measurement; for some respondents, the reason some measurements take longer is the desire to get a reliable reading. This included cleaning the child's digits or repositioning the probe:

"…taking longer does not mean that one doesn't know the procedure, but sometime it's because you want to give the correct reading." (CHW, Malawi)

A key challenge reported by frontline HCPs in Malawi was around keeping the oximeter charged; this was not considered a significant challenge in Bangladesh. However, here they had issues with ensuring the oximeter remained dry and protected during rains and being fully waterproof was desirable. Depending on usage, battery life was reported as 1 week – 2 months.

<u>Sustainability</u>

Sustainability was discussed in terms of the device's durability, and the need for continued professional development. Generally the pulse oximeters were thought of as robust and durable, with some of the HCPs having used their device for over four years without replacements. However, the battery was highlighted as the least durable part of the device, and there was a perception that when the battery was worn down the readings became less reliable.

"There is a matter with the battery too, if the battery is not enough the reading takes a long time to appear. It sometimes gives false negative readings." (Hospital, Bangladesh)

For peer review only - http://bmjopen.bmj.com/site/about/guidelines.xhtml

This related to the HCPs suggestion of having on-going maintenance support rather than wanting replacement devices. HCPs described the need for on-going training and support, but also expressed a desire for more in-depth education on how oximetry works which goes beyond the basic training to take a reliable measurement:

"A person gets used to what they are doing once they have been oriented. I think sometimes it's also good for you and your team to orient us on how this thing works [...] the way this thing works, we don't know" (Health centre, Malawi)

In terms of keeping the devices clean and properly stored, an important factor for prolonging shelf-life, we found conflicting opinions between Malawi and Bangladesh. Malawi deemed the probes easy to clean and store securely, although the light colour and materials of the device was thought to show dirt easily. However, in Bangladesh cleaning was described as burdensome; this likely reflects the different devices and therefore methods needed for cleaning, or different perceptions of the importance and frequency of cleaning.

"It is hard work to clean it with hexsol and cotton after coming back from the field every day. If we could get something else to clean it with so that we can clean once a week, I don't like cleaning it every day." (CHW, Bangladesh)

Design

The key challenges mentioned repeatedly across sites and HCP cadres were the battery, sensitivity of the probe in relation to movement or low perfusion, and the probe fit in younger children. Table 2 summarises the design features requested or suggested to improve the pulse oximeters for use in these low-resource settings. Suggestions covered the probe, such as having detachable probes of different sizes, charging and battery life, such as additional power packs and solar charging, and features to help with agitated children.

The oximeters which HCPs used were designed for continuous monitoring; therefore oxygen saturation is not a single static result. This was seen as a negative, with HCPs in both sites wanting the ability to stop at a result and even store measurements (e.g. a blood glucose monitor):

"Readings would fluctuate if the baby moves. We don't want that. After getting the actual reading it should stay fixed." (CHW, Bangladesh)

In Bangladesh specifically, the CHWs stated a preference for numbers or bars to indicate measurement quality, rather than a dynamic waveform display. This opinion was not reflected in Malawi, which could be a result of using different devices or different training. A specific challenge

For peer review only - http://bmjopen.bmj.com/site/about/guidelines.xhtml

presented by CHWs in Malawi was the use of the oximeter in direct sunlight; CHWs often hold clinics outside and they faced the combined challenges of bright sunlight and dust, both of which they reported as challenges in taking measurements.

"…it returns the correct results when you are in the shade, but while you are in sunlight it fails to determine good results." (CHW, Malawi)

Positive design features included the portability of devices, the ease of using them and perceived durability, with little direct criticism of the oximeters that the HCPs had been using:

"…of the things I like most about using the pulse oximeter, the first one is the portability, because I can use it anywhere." (Hospital, Malawi)

Discussion

We investigated end-user experiences of using pulse oximeters by a range of different HCPs across clinical settings in Malawi and Bangladesh. The FGDs highlighted similarities in experience, such as challenges in battery durability, the difficulty of small and agitated children and the positive impact of oximeters on clinical practice. However, there were key differences between the providers' experiences in Malawi and Bangladesh and between HCP cadres.

Of note was the difference in perceived ease of cleaning, which was seen as more burdensome in Bangladesh. This is likely associated with the Y-shaped wrap probe design, compared to the more easily cleaned clip design used in Malawi (Figure 1). Interestingly though, most critiques were similar between sites, highlighting some of the major challenges of using pulse oximetry in children – namely movement, low perfusion and small digits. This consistency between our sampled HCPs from each site suggests these challenges are not device dependent and therefore a specifically designed re-usable device for universal paediatric use in low-resource settings is needed.

We identified differences in the sense of value placed on the oximeters by the HCPs, with the higher trained HCPs attributing less value to the results than the HCPs with more limited training. Those with more training valued their clinical judgement more and were more willing to question the accuracy of SpO_2 results. This poses interesting lessons for scaling-up implementation and training, as despite perceptions that obtaining a SpO_2 measurement is generally easy, the interpretation of the result is more nuanced. Sustained mentorship and more in-depth training were desired by the HCPs, and this needs to be considered as part of any implementation programme.

For peer review only - http://bmjopen.bmj.com/site/about/guidelines.xhtml

As the oximeters were used as spot-check devices rather than continuous monitors, as would generally be found in operating theatres or high-dependency care in high-income settings, many of the suggested design changes related to improving the devices for this process. One example of this was the need for improved battery-life and charging, with HCPs highlighting their limited ability to easily access charging points, unlike high-income inpatient settings. Consistently, the desire for quicker, static results and a movement tolerant probe with improved fit on younger infants was important. Unexpected issues, such as usability in direct sunlight, emphasize the importance of end-user engagement in product development as clinical devices designed for high-income settings would not need to be robust to outdoor use.

The idea of a pulse oximeter being able to improve trust between a caregiver and healthcare provider poses potentially exciting opportunities for improving referral and treatment for paediatric pneumonia. Early diagnosis and treatment as downstream in the health system as possible, ideally to the level of CHWs, are key strategies for improving pneumonia outcomes and therefore reducing morbidity and mortality burden [25]. Therefore, an objective and simple clinical tool with in-built decision support, e.g. auditory or visual alarms when the SpO_2 is outside of normal range, presents an opportunity for caregiver education and empowerment in the referral decision-making process. Recent data from Malawi supports the potential for oximetry to improve referrals , with HCPs from frontline settings more than twice as likely to correctly refer clinically-eligible children with a SpO_2 <90% compared to those with a $SpO_2 \geq 90\%$ during routine outpatient care [12]. Interestingly, this has not necessarily been the case with other more objective diagnostic tools, with examples of rapid diagnostic malaria tests leading to provider-caregiver tensions around treatments [26,27].

This study was potentially subject to social-desirability bias, with healthcare workers expressing opinions which they thought the facilitators wanted to hear. The purpose of the study was explained to the participants during the consent process, and was highlighted as an opportunity for them to contribute to the design of a revised paediatric oximeter and probe. In addition, the groups in some cases were mixed in terms of gender and job titles, possibly influencing participant's confidence in expressing their views and experiences. To mitigate these potential biases, the facilitators encouraged the all participants to contribute to the discussions and to be critical throughout. Both positive and negative views were given in both Malawi and Bangladesh, and by different types of HCPs, therefore we do not feel these biases detract from our findings. Finally, we were limited by the number of groups we conducted; additional groups or a different sampling approach may have led to alternative perspectives being included, as the number was not driven by saturation. Therefore the conclusions we draw need to be interpreted accordingly.

For peer review only

Overall pulse oximeters were valued by the HCPs we sampled for this study, despite challenges with charging, maintenance and speed of achieving accurate readings in moving or smaller children. This implies that making improvements to currently available oximeters and probes could further facilitate successful implementation of this technology at the community through to the hospital level for routine paediatric care in these two settings. Based on these providers varied experiences, we recommend that efforts to re-design a pulse oximeter for paediatric spot-checks focus on improvements to battery durability, better fit for smaller digits and the speed at which readings are obtained; these were all important challenges which did not necessarily have local solutions presented. More substantive design changes could focus on alternative power and charging systems (e.g. solar charging) and '3-in-1' devices which include respiratory rate and temperature measurements.

For peer review only

Acknowledgments

We would like to thank all those healthcare providers who took part in the focus group discussions for their time. We would like to acknowledge other members of the study team: Katie Fernandez (Lifebox); Charles Makwenda and Tambosi Phiri (Parent and Child Health Initiative); Tim Colbourn (University College London); Mike Bernstein (Physio Monitor); and Nazma Begum, Arun Dutta Roy and Abu Abdullah Mohammad Hanif (Johns Hopkins University-Bangladesh).

Funding

This study was funded by the Bill & Melinda Gates Foundation (grant number: OPP1133291).

Conflict of Interest

Authors declare no conflicts of interest.

Author contributions

The qualitative study was designed and topic guides developed by IWi, IWa, CK and EDM, and the field manual written by CK. Oversight of the study was conducted by CK, BN and BZ in Malawi and EDM, AB and MI in Bangladesh. In Malawi, BZ and EK arranged, conducted, transcribed and translated the FGDs. In Bangladesh SA and MI arranged and conducted the FGDs. The data was coded and analysed by CK. The manuscript was written by CK, with considerable input from EDM. IWi, IWa, EDM, BZ, EK, SA, MI, NB, AB and BN read, commented and approved the manuscript.

Data Sharing Statement

Anonymised transcripts can be shared, following the signing of a data sharing agreement, subject to approval from the relevant National ethics committees. For further information please contact Dr. Carina King: c.king@ucl.ac.uk.

For peer review only

For peer review only - http://bmjopen.bmj.com/site/about/guidelines.xhtml

References

1. Sazawal S, Black RE, Pneumonia Case Management Trials G. Effect of pneumonia case management on mortality in neonates, infants, and preschool children: a meta-analysis of community-based trials. *Lancet Infect Dis* 2003;3(9):547-56.
2. Niessen LW, ten Hove A, Hilderink H, et al. Comparative impact assessment of child pneumonia interventions. *Bull World Health Organ* 2009;87(6):472-80.
3. Walker CL, Rudan I, Liu L, et al. Global burden of childhood pneumonia and diarrhoea. *Lancet* 2013;381(9875):1405-16. doi: 10.1016/S0140-6736(13)60222-6
4. Organisation WH. Oxygen therapy for children: a manual for health workers. Geneva: WHO 2016.
5. . WHO Recommendations on the Management of Diarrhoea and Pneumonia in HIV-Infected Infants and Children: Integrated Management of Childhood Illness (IMCI). Geneva2010.
6. Orimadegun A, Ogunbosi B, Orimadegun B. Hypoxemia predicts death from severe falciparum malaria among children under 5 years of age in Nigeria: the need for pulse oximetry in case management. *Afr Health Sci* 2014;14(2):397-407. doi: 10.4314/ahs.v14i2.16
7. Chhibber AV, Hill PC, Jafali J, et al. Child Mortality after Discharge from a Health Facility following Suspected Pneumonia, Meningitis or Septicaemia in Rural Gambia: A Cohort Study. *Plos One* 2015;10(9):e0137095. doi: 10.1371/journal.pone.0137095
8. Hooli S, Colbourn T, Lufesi N, et al. Predicting Hospitalised Paediatric Pneumonia Mortality Risk: An External Validation of RISC and mRISC, and Local Tool Development (RISC-Malawi) from Malawi. *Plos One* 2016;11(12):e0168126. doi: 10.1371/journal.pone.0168126
9. Wandeler G, Pauchard JY, Zangger E, et al. Which clinical signs predict hypoxaemia in young Senegalese children with acute lower respiratory tract disease? *Paediatrics and International Child Health* 2015;35(1):65-68. doi: 10.1179/2046905514Y.0000000153
10. Basnet S, Adhikari RK, Gurung CK. Hypoxemia in children with pneumonia and its clinical predictors. *The Indian Journal of Pediatrics* 2006;73(9):777-81. doi: 10.1007/bf02790384
11. Lodha R, Bhadauria PS, Kuttikat AV, et al. Can Clinical Symptoms or Signs Accurately Predict Hypoxemia in Children with Acute Lower Respiratory Tract Infections? *Indian Pediatr* 2003;41:129-35.
12. McCollum ED, King C, Deula R, et al. Pulse oximetry for children with pneumonia treated as outpatients in rural Malawi. *Bull World Health Organ* 2016;94:893-902. doi: http://dx.doi.org/10.2471/BLT.16.173401
13. Duke T, Subhi R, Peel D, et al. Pulse oximetry: technology to reduce child mortality in developing countries. *Ann Trop Paediatr* 2009;29(3):165-75. doi: 10.1179/027249309X12467994190011
14. Emdin CA, Mir F, Sultana S, et al. Utility and feasibility of integrating pulse oximetry into the routine assessment of young infants at primary care clinics in Karachi, Pakistan: a cross-sectional study. *BMC Pediatrics* 2015;15(1):141. doi: 10.1186/s12887-015-0463-z
15. King EM, Lieu C, Kasasa A, et al. Pulse Oximetry as a Screening Tool to Detect Hypoxia Associated with Early-onset Sepsis in Asymptomatic Newborns: A Feasibility Study in a Low-income Country. *British Journal of Medicine & Medical Research* 2014;4(5):1115-28.
16. Ginsburg AS, Izadnegahdar R, Klugman KP. World Pneumonia Day 2016: pulse oximetry and oxygen. *The Lancet Global Health* 2016;4(12):e893-e94. doi: http://dx.doi.org/10.1016/S2214-109X(16)30296-0
17. Ginsburg AS, Van Cleve WC, Thompson MI, et al. Oxygen and pulse oximetry in childhood pneumonia: a survey of healthcare providers in resource-limited settings. *J Trop Pediatr* 2012;58(5):389-93. doi: 10.1093/tropej/fmr103
18. Lifebox: Pulse Oximetry [Available from: http://www.lifebox.org/ accessed 23/11/2017.
19. The Phone Oximeter [Available from: http://www.phoneoximeter.org/ accessed 23/11/2017.
20. Spence H, Baker K, Wharton-Smith A, et al. Childhood pneumonia diagnostics: community health workers' and national stakeholders' differing perspectives of new and existing aids. *Glob Health Action* 2017;10(1):1290340. doi: 10.1080/16549716.2017.1290340

For peer review only - http://bmjopen.bmj.com/site/about/guidelines.xhtml

21. Nsona H, Mtimuni A, Daelmans B, et al. Scaling up Integrated Community Case Management of Childhood Illness: Update from Malawi. *Am J Trop Med Hyg* 2012;87(Suppl 5):54-60.
22. Malalwi Go. Integrated Management of Childhood Illness - Caring for Newborns and Children in the Community: Manual for Healt hSurveillance Assistants. In: Health Mo, ed. Lilongwe, Malawi, 2008.
23. Gale NK, Heath G, Cameron E, et al. Using the framework method for the analysis of qualitative data in multi-disciplinary health research. *BMC Medical Research Methodology* 2013;13(1):117. doi: 10.1186/1471-2288-13-117
24. Ritchie J, Spencer L. Qualitative data analysis for applied policy research. *The qualitative researcher's companion* 2002;573:305-29.
25. McCollum ED, King C, Hammitt LL, et al. Reduction of childhood pneumonia mortality in the Sustainable Development era. *Lancet Respir Med* 2016;4(12):932-33. doi: 10.1016/S2213-2600(16)30371-X
26. Ansah EK, Reynolds J, Akanpigbiam S, et al. "Even if the test result is negative, they should be able to tell us what is wrong with us": a qualitative study of patient expectations of rapid diagnostic tests for malaria. *Malaria Journal* 2013;12(1):258. doi: 10.1186/1475-2875-12-258
27. Hutchinson E, Reyburn H, Hamlyn E, et al. Bringing the state into the clinic? Incorporating the rapid diagnostic test for malaria into routine practice in Tanzanian primary healthcare facilities. *Glob Public Health* 2015:1-15. doi: 10.1080/17441692.2015.1091025

For peer review only

For peer review only - http://bmjopen.bmj.com/site/about/guidelines.xhtml

**Table 1:** Summary of the FGD participants and their work experience			
	Community level	Health centre or Upazila Health Complex	Hospital
**BANGLADESH**			
Total participants	8	7	8
Job titles (number)	Community healthcare worker (8)	Physician (4)<br>Medical officer (3)	Senior staff nurse (1)<br>Associate professor (2)<br>ICU staff (1)<br>Anaesthesiologist (1)<br>Assistant registrar (1)<br>Intern medical officer (1)
Work experience (mean, range)	1.7 years (0.6 – 4)	2.3 years (1 – 6)	14.7 years (0.5 – 32)
**MALAWI**			
Total participants	9	8	9
Job titles	Community healthcare worker (8)<br>Vital signs assistant (1)	Medical assistant (7)<br>Medical technician (1)	Clinical officer (3)<br>Nurse midwife (3)<br>Medical assistant (3)
Years' work experience (mean)	10.6 years (5 – 20)	8.3 years (3 – 23)	8.1 years (4 – 13)

For peer review only

For peer review only - http://bmjopen.bmj.com/site/about/guidelines.xhtml

Table 2: Suggestions of desirable features or improvements given by healthcare providers

**Challenge:**	**Design suggestion:**
Probe fit	Supplied with multiple sizes of probes for different ages<br>A single cable with multiple probes that can be changed (e.g. clipped into the cable)<br>Softer material for a more comfortable fit
Probe placement	Probe made of transparent material so sensor placement on the nail can be seen
Cleaning	Alcohol wipes provided for easier cleaning<br>Different colour probe to make it easier to see the dirt, but does not look dirty quickly
Power	Solar powered charger with rechargeable batteries<br>Back-up power bank<br>Supplied with a spare battery
Agitated children	Toy feature in the device to distract the child<br>Improve the sensitivity of the device to be quicker<br>Improve the sensitivity of the device to tolerate movement
Integrated spot-check device	Store results in a memory that can later be accessed<br>Static oxygen saturation result display<br>‘3-in-1’ device which includes temperature and respiratory rate measurements as well<br>Shorter cable length for easier portability

For peer review only

For peer review only - http://bmjopen.bmj.com/site/about/guidelines.xhtml

Figure legends:

Figure 1: Pulse oximeters and probe used by healthcare providers in routine clinical care

a. Lifebox® oximeter and adult universal clip probe used in Malawi (accessed on 1st July 2017 from: www.lifebox.org)
b. Masimo Rad5® oximeter and LNCS® Y-I Multisite wrap probe used in Bangladesh (accessed on 1st July 2017 from: www.pacificmedicalsupply.com)

For peer review only

For peer review only - http://bmjopen.bmj.com/site/about/guidelines.xhtml

a.

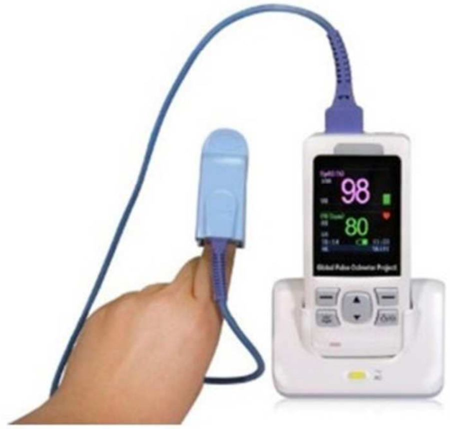

b.

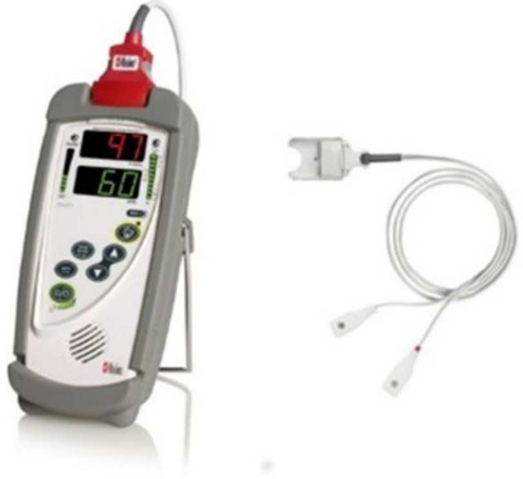

64x97mm (300 x 300 DPI)

Web Appendix 1 – Topic Guide

- What is your experience of using pulse oximeters in children?
- What have been the main issues you've encountered when using pulse oximeters?
- What have been the things you like most about using the pulse oximeters?
- What type of probes have you used? Have any been better than others? Why?
- Thinking about the probes, we would like to hear your feedback about some aspects of using them: ease of putting and keeping the probe on the child, durability, ease of taking a reading, ease of keeping it clean and storage
- Thinking about the oximeter, we would like to hear your feedback about some aspects of using them: ease of reading the display, durability, battery life and charging, time taken to get a reading
- What things would make the probe and pulse oximeter easier to use?
- What things would make the probe and pulse oximeter harder to use?

For peer review only

Appendix 2: Summary of pulse oximeter probes presented during focus group discussions with healthcare providers

Probe type	Figure	Product code
Neonatal wrap	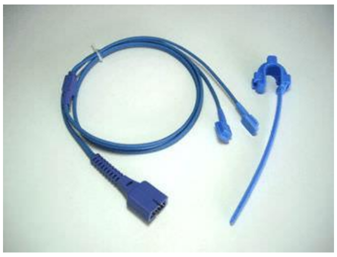	Acare ASYNR-D1
Adult clip	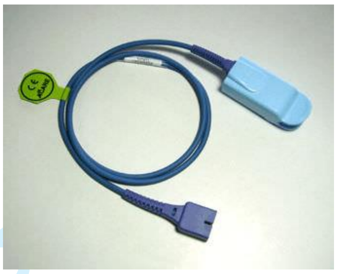	Acare ASANR-D1
Paediatric clip	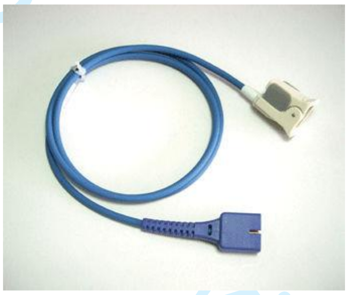	Acare ASPNR-D1
Ear clip	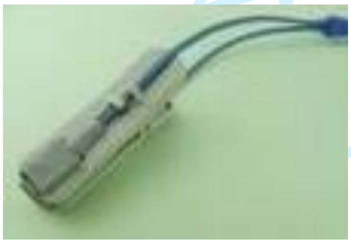	Nellcor U401-2HL
Adult boot	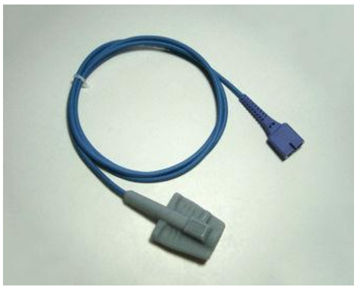	Acare ASSNR-D1
Paediatric boot	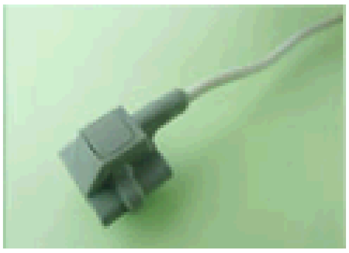	Nellcor U401-2EL

COREQ Checklist

No	Item	Guide questions/description	Page / evidence
Domain 1: Research team and reflexivity			
Personal Characteristics			
1.	Interviewer/ facilitator	Which author/s conducted the interview or focus group?	Pg 15, Author contributions
2.	Credentials	What were the researcher's credentials? *E.g. PhD, MD*	A mix of diploma, BSc, MSc and PhD
3.	Occupation	What was their occupation at the time of the study?	Pg 6, Methods: procedure
4.	Gender	Was the researcher male or female?	Both
5.	Experience and training	What experience or training did the researcher have?	Pg 6, 'Methods: Procedure'
Relationship with participants			
6.	Relationship established	Was a relationship established prior to study commencement?	No
7.	Participant knowledge of the interviewer	What did the participants know about the researcher? e.*g. personal goals, reasons for doing the research*	Pg 15, Discussion
8.	Interviewer characteristics	What characteristics were reported about the interviewer/facilitator? e.g. *Bias, assumptions, reasons and interests in the research topic*	Pg 15, Discussion
Domain 2: study design			
Theoretical framework			

For peer review only

No	Item	Guide questions/description	Page / evidence
9.	Methodological orientation and Theory	What methodological orientation was stated to underpin the study? *e.g. grounded theory, discourse analysis, ethnography, phenomenology, content analysis*	Pg 7, Methods: Analysis
Participant selection			
10.	Sampling	How were participants selected? *e.g. purposive, convenience, consecutive, snowball*	Pg 6, Methods: Sampling
11.	Method of approach	How were participants approached? e.*g. face-to-face, telephone, mail, email*	Pg 6, Sampling
12.	Sample size	How many participants were in the study?	Pg 7, Results and Table 1
13.	Non-participation	How many people refused to participate or dropped out? Reasons?	Pg 6, Sampling
Setting			
14.	Setting of data collection	Where was the data collected? e.*g. home, clinic, workplace*	Pg 6, Sampling
15.	Presence of non-participants	Was anyone else present besides the participants and researchers?	Pg 6, Methods: Procedure
16.	Description of sample	What are the important characteristics of the sample? *e.g. demographic data, date*	Table 1
Data collection			
17.	Interview guide	Were questions, prompts, guides provided by the authors? Was it pilot tested?	Appendix 1
18.	Repeat interviews	Were repeat interviews carried out? If yes, how many?	Not applicable

For peer review only

No	Item	Guide questions/description	Page / evidence
19.	Audio/visual recording	Did the research use audio or visual recording to collect the data?	Pg 7, Methods: Procedure
20.	Field notes	Were field notes made during and/or after the interview or focus group?	Pg 7, Methods: Procedure
21.	Duration	What was the duration of the interviews or focus group?	Between 1 and 2 hours
22.	Data saturation	Was data saturation discussed?	Pg 14, Discussion
23.	Transcripts returned	Were transcripts returned to participants for comment and/or correction?	This was not possible due to language potential barriers
Domain 3: analysis and findings			
Data analysis			
24.	Number of data coders	How many data coders coded the data?	Pg 7, Methods: Analysis
25.	Description of the coding tree	Did authors provide a description of the coding tree?	Pg 7, Methods: Analysis
26.	Derivation of themes	Were themes identified in advance or derived from the data?	Pg 7, Results
27.	Software	What software, if applicable, was used to manage the data?	Pg 7, Methods: Analysis
28.	Participant checking	Did participants provide feedback on the findings?	No, but discussed with local researchers (pg 7)

For peer review only

No	Item	Guide questions/description	Page / evidence
Reporting			
29.	Quotations presented	Were participant quotations presented to illustrate the themes / findings? Was each quotation identified? e.*g. participant number*	Throughout results section (pg 7 – 12)
30.	Data and findings consistent	Was there consistency between the data presented and the findings?	Throughout results section (pg 7 – 12)
31.	Clarity of major themes	Were major themes clearly presented in the findings?	Pg 7, Results
32.	Clarity of minor themes	Is there a description of diverse cases or discussion of minor themes?	Throughout results section (pg 7 – 12)

For peer review only
